# An Integrated Framework for Green Tea Infusion Chemical Characterization: Process Optimization, PCA-Based Chemometric Evaluation, and Untargeted Metabolomic Profiling

**DOI:** 10.3390/foods15203661

**Published:** 2026-10-15

**Authors:** Yi Bai, Wen-Ping Ma, Zhi-Jing Ni, Wei Wang, Kiran Thakur, Run-Hui Ma, Zhao-Jun Wei

**Affiliations:** 1 School of Biological Science and Engineering, North Minzu University, Yinchuan 750021, China; 2 Ningxia Key Laboratory of Development and Utilization of Specialty Food Resources, Yinchuan 750021, China

**Keywords:** green tea infusion, extraction optimization, target compounds, PCA comprehensive evaluation, non-targeted metabolomics

## Abstract

This study investigated the optimization and quality evaluation of green tea infusions prepared from three commercial green tea samples from different production origins, namely *Anhui Huangshan Maofeng* (AH), *Shaanxi Ziyang* (SX), and *Yunnan Xuelong* (YN), using three extraction techniques: hot-water extraction (HWE), enzyme-assisted extraction (EAE), and cold brewing (CB). We used water-extractable content (WEC) as the sole response variable in a response surface methodology framework to optimize physical solid yield rather than chemical quality. We developed a principal component analysis (PCA) model that incorporates WEC with six essential functional constituents—epicatechin (EC), epigallocatechin (EGC), epicatechin gallate (ECG), epigallocatechin gallate (EGCG), caffeine, and L-theanine, to thoroughly evaluate infusion quality. Untargeted metabolomics was subsequently performed on three PCA-selected representative SX samples corresponding to PCA ranks 18, 58, and 107. Our findings demonstrated that WEC was significantly influenced by the investigated extraction variables, and validation of the BBD-RSM models showed absolute relative deviations of 0.12–0.81% between the predicted and experimentally measured WEC values. Six targeted components differed significantly among the three tea samples and extraction methods. The PCA score plots showed partial separation among the sample–process combinations, with the first two principal components explaining 78.45% of the total variance. Additionally, the three SX infusion groups were clearly distinguished by orthogonal partial least-squares discriminant analysis (OPLS-DA) in conjunction with KEGG pathway mapping; KEGG pathway mapping shows that the differentially enriched metabolites are primarily annotated to flavonoid, phenolic acid, amino acid derivative, and purine-related pathways. By combining RSM-based optimization, targeted HPLC measurement, PCA scoring, and non-targeted metabolomic profiling, this study created a strong framework for chemical evaluation. This integrated framework offers a valuable reference for selecting appropriate extraction processes and ensuring consistent quality in green tea infusions.

## 1. Introduction

Green tea is an unfermented beverage made from the young shoots of the tea plant (*Camellia sinensis*). Catechins, caffeine, free amino acids, organic acids, and flavonoid derivatives are among the main non-volatile substances found in its infusion. They can differ according to the extraction conditions, processing technique, cultivar, and place of origin [1]. Catechins are among the main compounds that shape the taste and functional properties of green tea. Epigallocatechin gallate (EGCG), epigallocatechin (EGC), epicatechin (EC), and epicatechin gallate (ECG) mainly contribute to astringency, bitterness, and antioxidant activity [2]. L-theanine is closely related to freshness and a sweet aftertaste. Caffeine is a purine alkaloid that contributes to bitterness and the stimulating effect of tea [3]. Non-catechin flavonoids, such as flavone glycosides, are also associated with the sensory and functional properties of tea infusions [4].

Several process variables influence the extraction of tea infusion. These include temperature, particle size, extraction time, solid-to-liquid ratio, and the application of auxiliary extraction techniques. These elements work together to affect polyphenolic compound extraction efficiency and water-extractable content (WEC) [5]. Therefore, establishing a reliable extraction process requires the appropriate combination of these factors.

The conventional technique for making tea infusions is hot-water extraction (HWE). In recent years, additional extraction techniques have also been used. Enzymatic hydrolysis, pulsed electric fields, and ultrasonication can enhance the release of antioxidants, polyphenols, and catechins from green tea. By disrupting vacuoles containing active compounds and electroporating the cell wall, PEF-assisted extraction can increase extraction efficiency [6]. The release of catechins and their contribution to bioactivity can also be influenced by temperature and the extraction medium [7]. Because cold brewing is done at a lower temperature than HWE, it offers a different extraction pattern. The development of fragrance, taste, and color as well as the release of flavor chemicals are all impacted by this process. Variations in several metabolites are linked to these alterations. Cold brewing at 4 °C decreased the extraction of bitter compounds like caffeine by more than 40%, according to a recent metabolomics study. When compared to hot-brewed tea, the same treatment raised L-theanine by roughly 9.2% [8].

Both process rationality and component recovery demonstrate the value of an extraction process. Therefore, a process should be assessed using its entire chemical makeup rather than just one yield measure. Total extraction yield, total phenolic content, antioxidant activity, or a single target molecule were frequently used as the primary response variable in earlier research on the extraction of tea and plant bioactive compounds [9]. Therefore, WEC was used in the present study as a practical response variable for optimizing the extraction of total water-extractable matter. However, WEC alone cannot represent the overall chemical or sensory quality of a tea infusion. For this reason, HPLC-based targeted analysis and PCA were subsequently used to characterize differences in selected chemical constituents among the samples. Phenolic acids, caffeine, fatty acids, and amino acids in matcha and other tea infusions can reflect tea quality from different chemical dimensions [10]. Accordingly, in the present study, WEC was used only as an indicator of physical solid yield for RSM optimization and was not considered a measure of optimized chemical quality. Chemical characteristics were subsequently evaluated separately using targeted chemical analysis and multivariate approaches.

Establishing a comprehensive evaluation system for tea extracts requires advanced analytical methods to characterize the complex chemical profiles of tea extracts and the patterns of variation exhibited under different processing conditions. Tea constituents such as catechins, caffeine, and L-theanine are commonly characterized using chromatographic and mass-spectrometric techniques, including HPLC and LC-MS [11]. Metabolomics has also become a systematic tool for elucidating the complex chemical changes during tea processing and extraction. Broad-range targeted and untargeted metabolomics have been used to reveal the dynamic changes in key flavor metabolites during the processing of Longjing green tea and to analyze the effects of different withering levels on non-volatile metabolites and sensory characteristics [12]. Relevant reviews indicate that metabolomics combined with multivariate statistics can be used to identify key metabolites during tea growth, processing, and storage [13]. In various tea infusions, matcha, and green tea aroma systems, the combination of metabolomics and chemometrics has also demonstrated its applicability in distinguishing sample types and elucidating flavor differences [14]. Furthermore, non-targeted metabolomics in food science has been widely used for authenticity identification, differential metabolite screening, and pathway analysis in complex matrices [15].

Previous studies on green tea extracts have generally examined individual processing approaches, restricted sets of indicators, or a single type of raw material, while studies integrating WEC-based response surface optimization, HPLC-based targeted quantification, PCA-based comprehensive chemical scoring, and homogeneous non-targeted metabolomics remain scarce. Multivariate methods such as PCA are commonly used to construct comprehensive food quality evaluation systems [16], and non-targeted metabolomics has also been shown to provide systematic evidence for the screening of differentially abundant metabolites and the analysis of metabolic pathways in tea systems [17]. Changes in aroma, volatile compounds, and sensory-related metabolites during green tea processing can be characterized using targeted and metabolomics-based techniques [18,19]. Therefore, this study focused on three green tea samples, namely *Anhui Huangshan Maofeng* (AH), *Shaanxi Ziyang* (SX), and *Yunnan Xuelong* (YN), to compare the differences in WEC and targeted chemical composition among three extraction processes:HWE, enzyme-assisted extraction (EAE), and cold brewing (CB). We used a PCA-based comprehensive chemical score to select representative samples from the same SX tea source for untargeted metabolomics analysis. Similar integration of multivariate analysis with targeted and untargeted metabolomics has been applied to characterize chemical variation among representative tea samples [20]. Accordingly, the metabolomics analysis aimed to further characterize differences in the chemical profiles of tea infusions under different extraction processes.

## 2. Materials and Methods

### 2.1. Materials and Instruments

The *Anhui Huangshan Maofeng* (AH), *Shaanxi Ziyang* (SX), and *Yunnan Xuelong* (YN) used in this experiment were purchased from Huangshan Huixiaosheng E-commerce Co., Ltd. (Huangshan, China), Ziyang County Runyuan Tea Co., Ltd. (Ziyang, China), and Anji Xilong Yurui Tea Farm (Anji, China), respectively. Each tea represented a single commercial batch from its respective source; cultivar, harvest, grade, and manufacturing-related factors were not independently controlled in the present experimental design. The tea leaves were dried, ground, and sieved into different particle sizes; the tea powders of various particle sizes were then stored in sealed containers at low temperatures, protected from light, and set aside for later use.

Acetonitrile, methanol, and phosphoric acid (chromatographic grade) were all purchased from Tianjin Saifurui Chemical Handan Co., Ltd (Tianjin, China). EGC (purity 98% by HPLC, CAS 970-74-1), EC (98%, CAS 490-46-0), EGCG (98%, CAS 989-51-5), ECG (98%, CAS 1257-08-5), caffeine (98%, CAS 58-08-2), and L-theanine (98%, CAS 3081-61-6) standards were purchased from Nanjing Yuanzhi Biotechnology Co., Ltd (Nanjing, China). Cellulase (100,000 U/g; Lot No. 3200251026) was purchased from Shandong Longkete Enzyme Preparation Co., Ltd. (Xishui, China), and pectinase (50,000 U/g; Lot No. KS385208) was purchased from Shanghai Yuanye Biotechnology Co., Ltd (Shanghai, China).

Agilent LC1200 High-Performance Liquid Chromatograph (Santa Clara, CA, USA), HH W-2 Constant-Temperature Water Bath (Shanghai, China), UPT-III-20 Ultrapure Water System (Santa Clara, CA, USA), LC-GMP-15 Vacuum Pump (Changzhou, China), LC-FAG-5000B Sand Core Filter Unit (Changzhou, China), DW-86L386 Ultra-Low Temperature Freezer (Changzhou, China), CT15RT Low-Temperature High-Speed Centrifuge (Shanghai, China), IMS-30 Fully Automatic Flake Ice Maker (Changshuo, China).

### 2.2. Experimental Methods

#### 2.2.1. Preparation of Tea Extract

The HWE treatment involved extraction with deionized water at 95 °C. The EAE treatment was based on the composite enzyme-assisted extraction method for tea established by Oanh et al. [21], the EAE treatment was performed using a composite enzyme preparation consisting of cellulase and pectinase at a mass ratio of 1:1. The declared activities of cellulase and pectinase were 100,000 U/g and 50,000 U/g, respectively. The total enzyme dosage was 0.003 g per gram of tea powder. The extraction medium was adjusted to pH 5.0 using a disodium hydrogen phosphate–citric acid buffer, and the extraction temperature was maintained at 50 °C. Extraction time was treated as an experimental variable and was set according to the corresponding single-factor or Box–Behnken experimental design. After extraction, the samples were placed in a boiling-water bath for 10 min to inactivate the enzymes. Extraction using deionized water at 20 °C was part of the CB treatment. The solid-to-liquid ratio, extraction time, and particle size were all investigated in the single-factor studies. Following extraction, the tea extract was cooled to room temperature, filtered through filter paper, and kept at −20 °C for later WEC, specific component analysis using HPLC, and thorough chemical analysis. The tea sample designation and extraction method were used to code the samples.

#### 2.2.2. Optimization of Extraction Conditions Based on WEC

This study assessed the extraction levels of soluble compounds in tea infusions using WEC as the response variable. WEC was determined according to GB/T 8305-2013 (Tea—Determination of water extracts content, Beijinng, China, 2013) and expressed on a dry-matter basis as follows:

(1) $$\mathrm{WEC}\;\left(\%\right)=\left[1-\frac{m_{1}}{m_{0}\times w}\right]\times 100$$

where m_0_ is the mass of the tea powder sample (g), m_1_ is the mass of the dried tea residue after extraction (g), and w is the dry-matter mass fraction of the tea powder. Single-factor trials were first used to assess the effects of solid-to-liquid ratio (A), extraction time (B), and particle size (C) on WEC and to define the factor ranges used in the subsequent response surface design. WEC was used as the sole response variable in the BBD-RSM analysis. Therefore, the optimization aimed specifically at maximizing WEC and was not intended to determine the overall optimum for chemical composition or sensory quality. Based on the single-factor experiments, a three-factor, three-level Box–Behnken design (BBD) was used to model WEC as a function of solid-to-liquid ratio (A), extraction time (B), and particle size (C). The coded and actual levels of the three factors are provided in Table S2. A quadratic polynomial model was fitted separately for each of the nine tea sample–process combinations. The complete regression equations and the corresponding WEC-optimized conditions are provided in Table S5, while the full ANOVA results for the models and individual model terms are presented in Table S3. WEC was the sole response variable used for optimization; therefore, the resulting conditions are referred to as WEC-optimized conditions. The predicted WEC values were subsequently verified experimentally.

#### 2.2.3. HPLC Quantitative Analysis of Selected Target Compounds in Green Tea Infusion

The concentrations of EGC, EC, EGCG, ECG, caffeine, and L-theanine were determined by HPLC using the external-standard method; no internal standard was used. Concentrations expressed as mg/g were calculated per gram of tea powder used for extraction. The chromatographic analysis of catechins and caffeine was adapted from a previously reported HPLC method for quantifying tea catechins and caffeine, with appropriate modifications according to the tea infusion matrix and chromatographic system used in this study [22]. Prior to injection, tea infusion samples were passed through a 0.45 μm microporous membrane. EGC, EC, EGCG, ECG, and caffeine were analyzed using an Agilent 1260 Infinity HPLC equipped with a diode array detector (DAD) and a C18 reverse-phase column (250 mm × 4.6 mm, 5 μm; Agilent Technologies, Santa Clara, CA, USA). Ultrapure water (phase A), 50 mmol/L aqueous phosphoric acid (phase B), and acetonitrile (phase C) made up the mobile phase. The column was maintained at 35 °C; 20 μL of each sample was injected at a flow rate of 0.8 mL/min and monitored at 280 nm. The gradient elution program was as follows: 0 min, A/B/C = 5/82/13 (*v*/*v*/*v*); 22 min, A/B/C = 9/70/21 (*v*/*v*/*v*); and 28 min, A/B/C = 25/50/25 (*v*/*v*/*v*).

The method for measuring L-theanine was taken from Ayakdaş and modified appropriately to take into consideration the tea infusion sample matrix and instrument conditions [3]. The mobile phase consisted of methanol (Phase A) and a 0.5% aqueous phosphoric acid solution (Phase B). Isocratic elution was employed, with the A/B ratio maintained at 10/90 (*v*/*v*) over a 0–25 min period. The flow rate was 0.8 mL/min, the column temperature was 25 °C, the injection volume was 20 μL, and the detection wavelength was 210 nm. Tea infusion samples were filtered through a 0.45 μm microporous filter membrane before injection for analysis.

See Table S8 for the HPLC method validation.

#### 2.2.4. Comprehensive Chemical Scoring of Green Tea Infusions Based on PCA

With appropriate modifications to the approach of Zhang et al. [16], PCA was applied to characterize multivariate chemical differences among the green tea infusions. PCA variables included WEC, EGC, EC, EGCG, ECG, caffeine, and L-theanine. All variables were standardized prior to PCA. For the composite scoring procedure, caffeine was treated as an inverse-oriented variable and was directionally transformed before standardization as follows:

*X_b_* = *X*_max_ − *X_i_* (2)

In Formula (2), *X_b_* represents the directionally transformed caffeine value of the ith sample; *X_i_* represents the original caffeine content of the ith sample; and *Xmax* represents the maximum caffeine content among all samples. The transformed caffeine values were subsequently standardized together with the other variables using Z-score standardization.

Duplicate records corresponding to identical experimental conditions were removed before PCA, and each unique experimental condition was treated as one observation. The comprehensive chemical score was calculated by weighing the variance contribution of each retained principal component:

(3) $$F=\sum_{i=1}^{n}\mathrm{aiFi}$$

In Formula (3), F represents the comprehensive chemical score; Fi represents the score for the i-th principal component; ai represents the ratio of the variance contribution of the i-th principal component to the cumulative variance contribution of the retained principal components; and *n* represents the number of retained principal components. This score was used as an exploratory descriptor for comparing multivariate chemical characteristics among tea infusion samples and for selecting representative samples. The explained-variance-based weights reflect the variance captured by each principal component rather than sensory or functional desirability.

#### 2.2.5. Classification of Green Tea Infusion Samples Based on a Comprehensive Chemical Score Derived from PCA

All tea infusion samples were ranked according to their PCA-derived composite chemical scores and divided into high-, medium-, and low-score regions. The high-, medium-, and low-score groups represent only differences in the exploratory composite chemical scores calculated from WEC and the six targeted chemical components and do not represent sensory quality grades. To minimize the influence of tea origin, representative samples were selected only from the SX tea samples. The samples at PCA ranks 18, 58, and 107 were selected from the high-, medium-, and low-score regions, respectively. These samples corresponded to S-F-S-M-4, S-M-S-L-1, and S-L-S-L-1. The PCA ranking was used only for representative sample selection and was not used to identify RSM-optimized samples.

#### 2.2.6. Screening of Representative Homologous SX Tea Samples for Non-Targeted Metabolomics

To minimize interference from different tea sources in the unscreened metabolomics comparison, this study selected only SX green tea samples from the same source for unscreened metabolomics analysis. Six biological replicates were included in each group (n = 6), resulting in 18 samples in total. Three representative SX samples selected from the PCA ranking were used for untargeted metabolomics analysis. Group A corresponded to PCA rank 18 (SX-HWE-R18), which was prepared by HWE at 95 °C with a solid-to-liquid ratio of 1:150 (g:mL), an extraction time of 45 min, and a particle size of 80 mesh. Group B corresponded to PCA rank 58 (SX-EAE-R58), which was prepared by EAE at 50 °C with a solid-to-liquid ratio of 1:90 (g:mL), an extraction time of 45 min, and a particle size of 60 mesh. Group C corresponded to PCA rank 107 (SX-CB-R107), which was prepared by CB at 20 °C with a solid-to-liquid ratio of 1:90 (g:mL), an extraction time of 2 h, and a particle size of 60 mesh. After the representative nodes had been selected, tea infusions were freshly prepared according to the exact conditions of the corresponding experimental nodes and submitted for LC-MS/MS analysis. These samples were representative single-factor experimental nodes and were not the corresponding RSM-optimized samples. The sample relationships used in the metabolomics analysis are shown in Table S1.

#### 2.2.7. Non-Targeted Metabolomics Analysis

The non-targeted metabolomics analysis was performed by Wuhan Maiwei Metabolomics Biotechnology Co., Ltd. (Wuhan, China). Tea infusion samples were stored at −80 °C and vortexed to homogenize after thawing. A 200 μL sample was mixed with an equal volume of 70% methanol extraction solution containing an internal standard. After vortex extraction for 15 min, the mixture was centrifuged at 4 °C and 12,000 r/min for 3 min. The supernatant was filtered through a 0.22 μm microporous filter membrane and used for LC-MS/MS analysis. Quality control (QC) samples were prepared by mixing equal volumes of each sample, and one QC sample was inserted every 10 samples during the analysis to evaluate instrument stability and data reproducibility. Chromatographic analysis was performed using a Shimadzu LC-30A ultra-high-performance liquid chromatography (UHPLC) system (Suzhou, China) and a Waters ACQUITY UPLC HSS T3 column (2.1 mm × 100 mm, 1.8 μm; Milford, MA, USA). Ultrapure water and acetonitrile, each containing 0.1% formic acid, were used as mobile phases A and B, respectively. Chromatographic separation was performed at 40 °C with a flow rate of 0.40 mL/min and an injection volume of 4 μL. The gradient elution program was as follows: 0–5 min, phase B increased from 5% to 65%; 5–6 min: Phase B increased to 99%; 6–7.5 min: held at 99%; 7.5–7.6 min: decreased to 5%, and equilibrated to 10 min. Mass spectrometry detection was performed using an AB SCIEX TripleTOF 6600+ system (Framingham, MA, USA), with data acquired in both positive and negative ion modes via electrospray ionization. The ion source temperature was 550 °C, with ion spray voltages of 5000 V and −4000 V, respectively; the MS^1^ and MS^2^ scan ranges were m/z 50–1250 and 25–1250, respectively.

The raw data were converted to mzML format using ProteoWizard. XCMS was used for peak extraction, peak alignment, and retention time correction, and missing values and signal drift were corrected using K-nearest neighbors interpolation and support vector regression. Metabolites were annotated based on the laboratory’s in-house database, public databases, predictive databases, and metDNA; only metabolites with a comprehensive identification score > 0.5 and a QC sample CV < 0.5 were retained. After log_2_ transformation and unit-variance scaling, PCA and OPLS-DA were used to evaluate metabolic differences among samples, and 200 permutation tests were performed to assess model robustness. Differentially abundant metabolites were screened using the criteria of FC ≥ 2 or FC ≤ 0.5, VIP > 1, and *p* < 0.05. No multiple-testing correction was applied; therefore, the *p*-value threshold was used as an exploratory screening criterion. These metabolites were then subjected to further enrichment analysis.

### 2.3. Statistical Analysis

Each experimental condition was independently extracted three times (n = 3), and each independently prepared tea infusion was treated as an experimental unit. Results are expressed as mean ± standard deviation. Data processing and statistical analysis were performed using SPSS version 24, Origin version 2025, Design-Expert version 13, and SIMCA version 14.1. Data normality and variance homogeneity were evaluated by the Shapiro–Wilk and Levene tests, respectively. Group means were compared by one-way ANOVA followed by Tukey’s HSD test, with significance defined at *p* < 0.05.

## 3. Results

### 3.1. Effect of Single-Factor Process Conditions on Water-Extractable Content (WEC)

**Figure 1:**
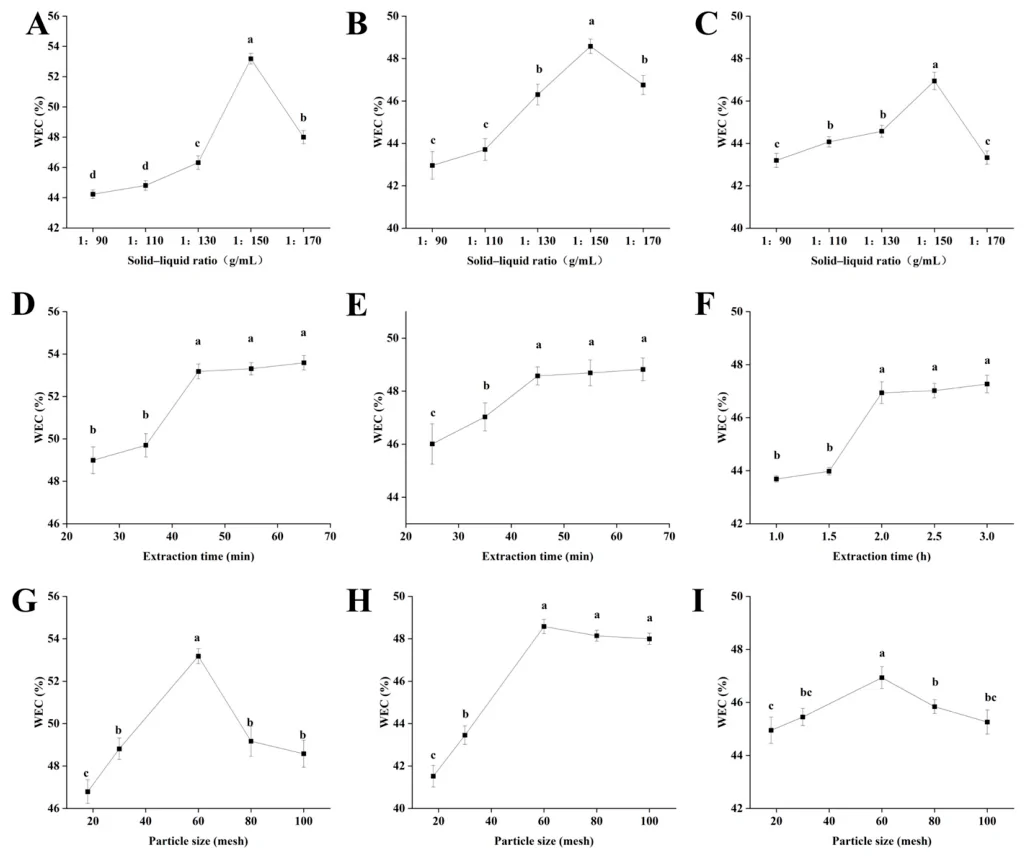
Single-factor optimization of extraction conditions for WEC in SX green tea infusions. (**A**–**C**) WEC at different solid-to-liquid ratios under HWE, EAE, and CB, respectively. (**D**–**F**) WEC at different extraction times under HWE, EAE, and CB, respectively. (**G**–**I**) WEC at different particle sizes under HWE, EAE, and CB, respectively. Different lowercase letters indicate significant differences among groups within the same factor (*p* < 0.05).

The effects of the solid-to-liquid ratio, extraction time, and particle size on WEC under the three extraction techniques were investigated using single-factor tests. SX tea exhibited comparable WEC patterns under HWE, EAE, and CB, as seen in Figure 1.

WEC visibly increased when the solid-to-liquid ratio rose from 1:90 to 1:150. At 1:150, the greatest values under HWE, EAE, and CB were 53.18%, 48.58%, and 46.94%, respectively. WEC dropped to 48.00%, 46.75%, and 43.33%, respectively, when the ratio rose to 1:170.Extraction time showed a similar pattern. WEC increased at first and then became stable. At 45 min, WEC under HWE and EAE was 53.18% and 48.58%, respectively. After this point, the increase became slower. Under CB, WEC reached 46.94% at 2 h and then showed a similar plateau. Particle size also affected WEC. At 60 mesh, the three treatments yielded 53.18%, 48.58%, and 46.94%, respectively. Finer particles caused a consistent decrease in WEC across all treatments. The extraction patterns of AH and YN were broadly consistent with those of SX (Figures S1 and S2).

### 3.2. WEC Optimization by Response Surface Methodology and Model Validation

**Figure 2:**
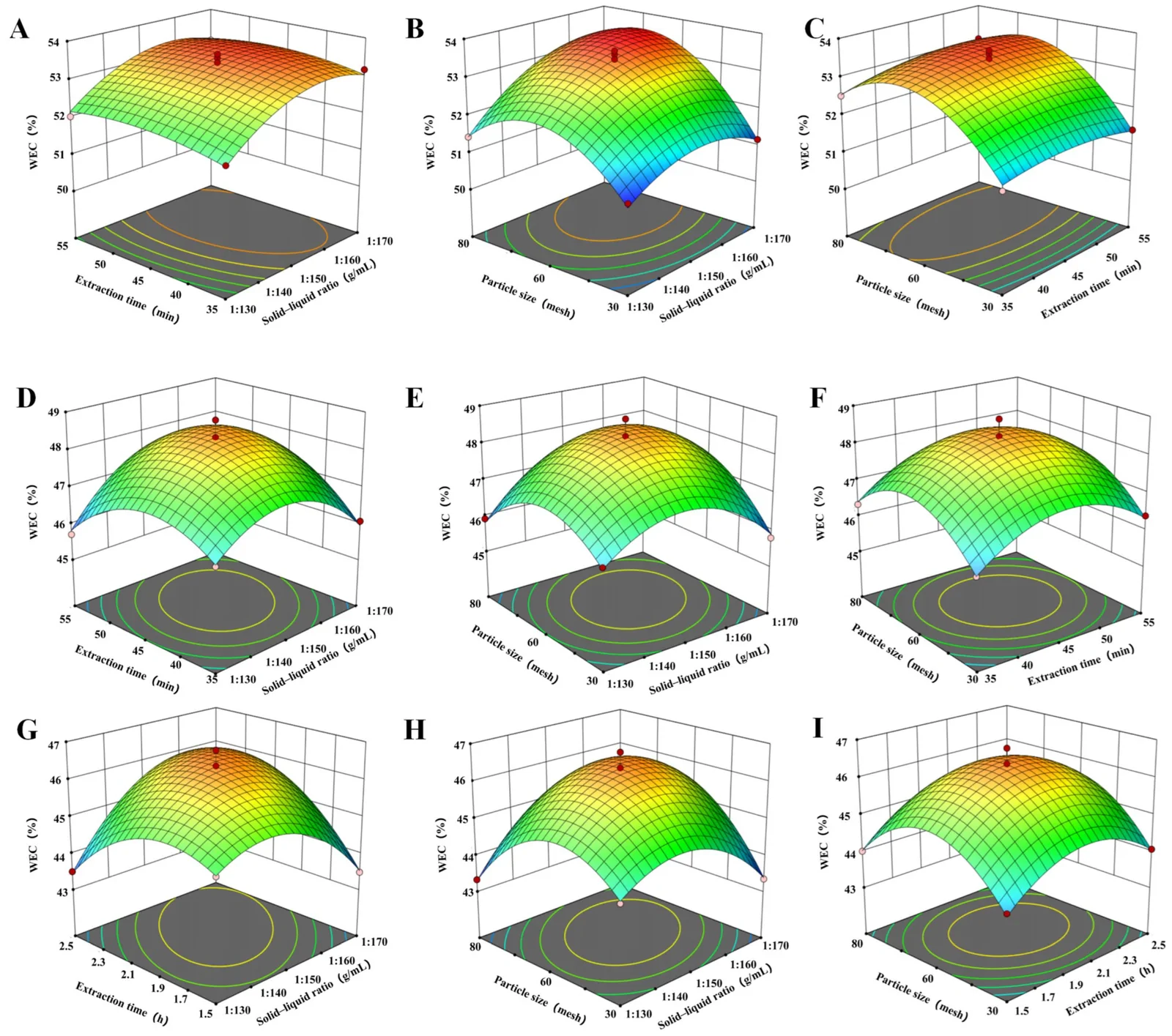
Three-dimensional response surface plots showing the effects of extraction variables on WEC in SX green tea infusions. (**A**–**C**), (**D**–**F**), and (**G**–**I**) represent HWE, EAE, and CB, respectively. Within each extraction process, the plots show the interactions between solid-to-liquid ratio and extraction time, solid-to-liquid ratio and particle size, and extraction time and particle size, respectively. The corresponding two-dimensional contour plots are provided in the Supplementary Materials.

A Box-Behnken design (BBD) was employed to optimize the extraction conditions for AH, SX, and YN teas. The quadratic polynomial models for all nine tea sample–process combinations were statistically significant (*p* < 0.001), whereas the lack-of-fit tests were non-significant (*p* > 0.05). The complete ANOVA results, including SS, df, MS, F-values, and *p*-values for the overall models and the individual linear, interaction, and quadratic terms, are provided in Table S3, while the model fit ststistics are presented in Table S4. The R^2^ values ranged from 0.9638 to 0.9877, the adjusted R^2^ values from 0.9172 to 0.9720, and the predicted R^2^ values from 0.7931 to 0.9656. The coefficients of variation ranged from 0.2722% to 0.6555%, and the adequate precision values were all greater than 14. The corresponding regression equations and WEC-optimized conditions for the nine models are provided in Table S5. The response surface and contour plots showed interactions among the solid-to-liquid ratio (A), extraction time (B), and particle size (C).WEC was impacted by these interactions to varying degrees. The significance of the linear, interaction, and quadratic terms varied among the nine models, indicating that the effects of the extraction variables and their interactions depended on the tea sample and extraction process. The F-values and *p*-values for all individual model terms are provided in Table S3. Figure 2 shows the nine 3D response surface plots for SX green tea, while the corresponding 2D contour plots are provided in the Supplementary Materials. The corresponding response surface results for AH and YN are also provided in the Supplementary Materials. WEC was generally higher near the central region of the design space. This result supported the next step of parameter optimization.

To validate the BBD-RSM models, WEC was experimentally determined under the WEC-optimized conditions for all nine tea sample–process combinations. The absolute relative deviations between the predicted and experimental WEC values were 0.81%, 0.14%, 0.59%, 0.35%, 0.56%, 0.41%, 0.12%, 0.21%, and 0.51% for AH-HWE, AH-EAE, AH-CB, SX-HWE, SX-EAE, SX-CB, YN-HWE, YN-EAE, and YN-CB, respectively. All deviations were below 1.0%, indicating generally close agreement between the model predictions and experimental measurements within the investigated design space. Confirming that the established models adequately predicted WEC under the conditions optimized with respect to this response variable (Table S6).

### 3.3. Effects of Extraction Conditions on Major Bioactive Compounds

**Figure 3:**
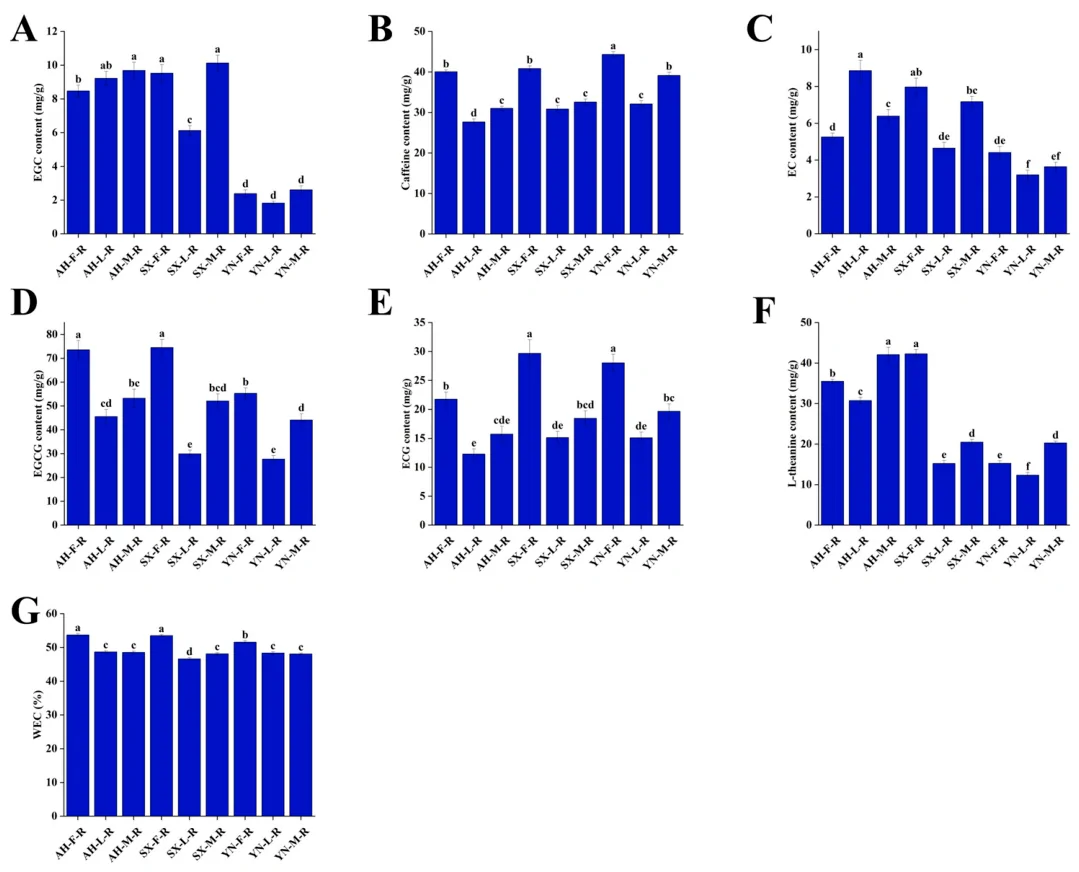
Chemical composition of green tea infusions prepared under WEC-optimized extraction conditions. (**A**) EGC; (**B**) caffeine; (**C**) EC; (**D**) EGCG; (**E**) ECG; (**F**) L-theanine; and (**G**) WEC. Different lowercase letters above the bars indicate significant differences among sample–process combinations for the same indicator according to Tukey’s HSD test (*p* < 0.05).

Under the optimized WEC conditions, HPLC analysis revealed significant differences in the concentrations of the six target compounds across sample–process combinations according to Tukey’s HSD test (*p* < 0.05) (Figure 3). EGCG was the most abundant catechin. Its level was highest in the AH-HWE and SX-HWE groups, whereas lower levels were observed in the CB-treated groups, particularly SX-CB and YN-CB. ECG and EC also varied among tea samples and extraction methods. EGC showed relatively high levels in AH-EAE, SX-HWE, and SX-EAE, while the YN groups showed lower levels overall. Caffeine content differed greatly among the samples, with YN-HWE showing the highest level, followed by AH-HWE, SX-HWE, and YN-EAE. L-theanine was highest in the AH-EAE and SX-HWE groups, while relatively lower levels were observed in the YN treatments. The complete WEC and HPLC target compound data under optimized conditions are shown in Table S7.

Table S8 shows the validation results for the HPLC method. The six target compounds showed good linearity, with R^2^ values ranging from 0.9964 to 1.0000. Across the six analytes, LODs were 0.0006–0.0028 mg/mL and LOQs were 0.00182–0.00848 mg/mL. The RSD values for precision, repeatability, and stability were all below 5%. The recovery rates ranged from 97.49% to 103.37%. These results showed that the method was accurate and stable for quantifying the six target compounds.

**Figure 4:**
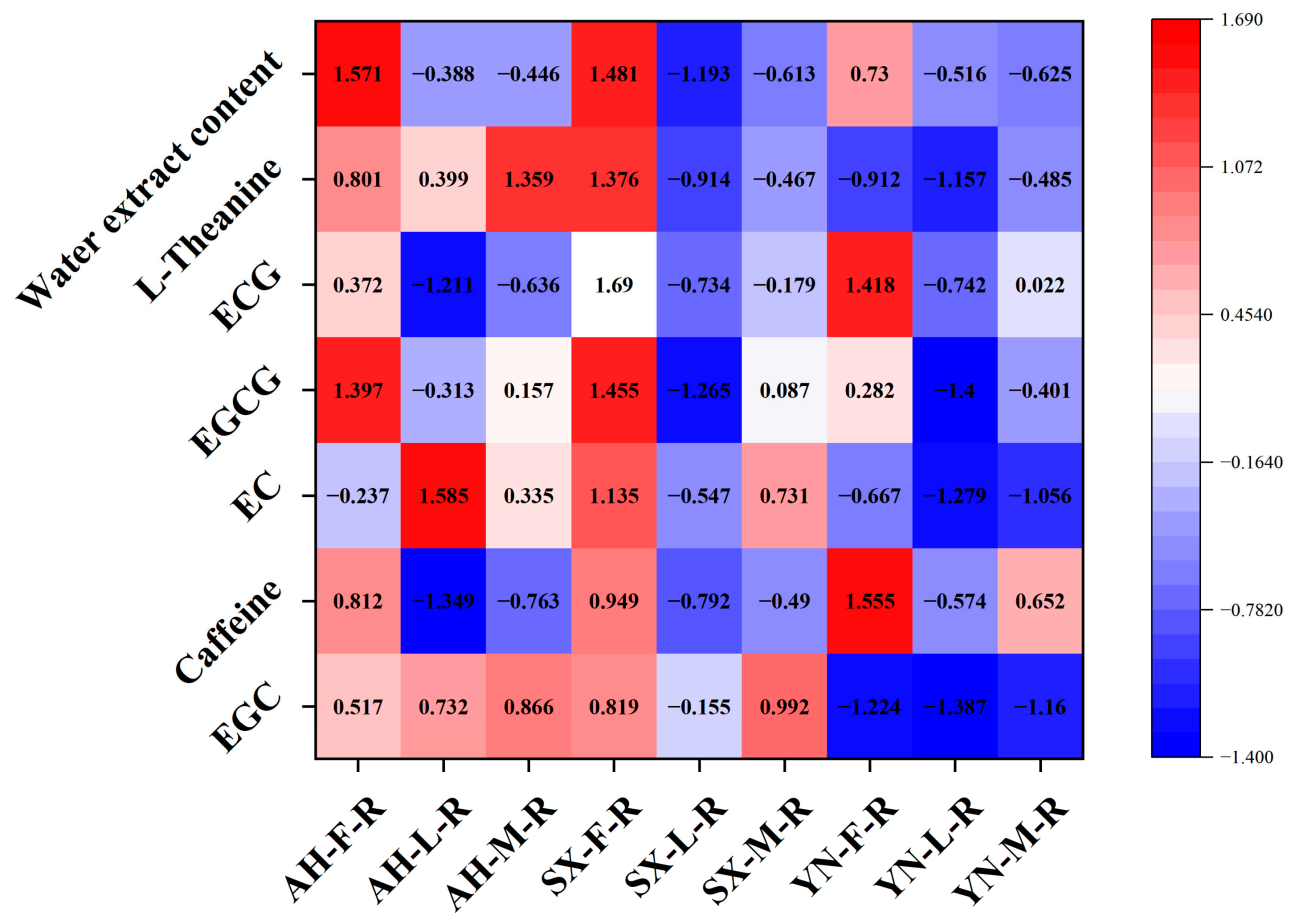
Heatmap of WEC and HPLC-targeted compounds in green tea infusion under optimized extraction conditions. The color scale represents column-wise Z-scores.

After column-wise Z-score standardization, Figure 4 showed the chemical profiles of different sample–process combinations. The standardization was performed separately for WEC and each of the six HPLC-targeted compounds using their mean values across the nine sample–process combinations, thereby allowing indicators with different units and numerical ranges to be compared on a common scale. AH-HWE and SX-HWE showed higher levels of WEC, EGCG, and L-theanine. SX-HWE had the strongest enrichment of EGCG and L-theanine. In contrast, YN-CB and YN-EAE showed lower levels of EGC, EC, and other catechin-related indicators. This pattern suggests that YN tea had a different chemical profile from AH and SX. Caffeine showed moderate differences among the sample-process combinations. This result indicates that alkaloids and catechins were not released in the same pattern during extraction.

### 3.4. Comprehensive Chemical Evaluation Based on PCA

**Figure 5:**
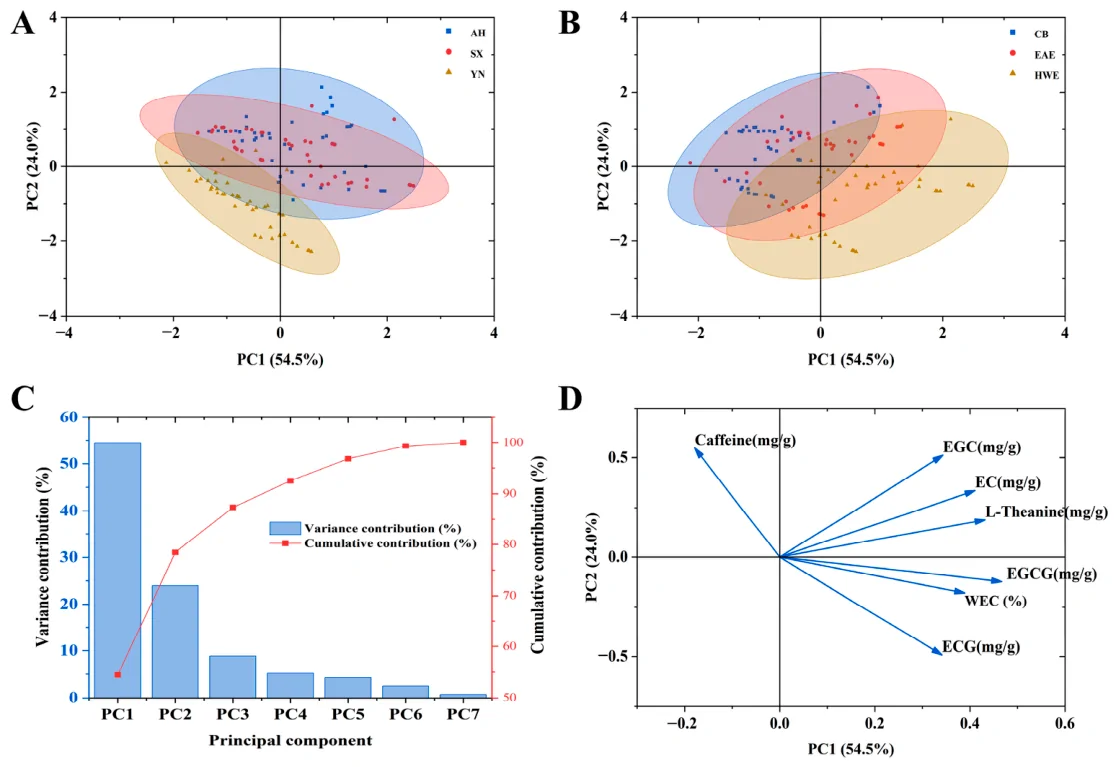
PCA-based comprehensive chemical evaluation of green tea infusions based on WEC and HPLC-targeted compounds. (**A**,**B**) PCA score plots grouped by tea origin and extraction process, respectively; (**C**) variance contribution and cumulative contribution of principal components; (**D**) loading plot of WEC and HPLC-targeted compounds.

To holistically evaluate the chemical characteristics of the different experimental conditions, PCA was performed using the standardized values of WEC and the six HPLC-targeted components after removal of duplicated center-point records (Table S9). A total of 126 unique experimental conditions were included in the PCA. Two principal components with eigenvalues > 1 were retained, PC1 and PC2 accounted for 54.47% and 23.98% of the total variance, respectively, giving a cumulative explained variance of 78.45% (Figure 5C). The score plots (Figure 5A,B) showed that samples from different origins and extraction processes were partially separated in the PC1/PC2 space; notably, HWE samples were predominantly distributed in the positive region of PC1, while CB samples clustered toward the negative region, indicating that the extraction method strongly influenced the chemical differentiation of the infusions. The loading plot (Figure 5D) revealed that EGCG, L-theanine, EC, EGC, and WEC loaded positively on PC1, whereas caffeine showed a stronger positive correlation with PC2. Detailed loadings and score coefficients are provided in Table 1.

A comprehensive chemical score (F) was calculated based on PC1 and PC2. The 126 unique experimental conditions were ranked according to their F values and equally divided into high-, medium-, and low-score zones (Table S10). S-F-S-M-4 ranked 18th in the high-score zone, S-M-S-L-1 ranked 58th in the medium-score zone, and S-L-S-L-1 ranked 107th in the low-score zone. These three representative SX samples were used for untargeted metabolomics analysis. Among all samples, S-F-R had the highest composite score (F = 1.8652), indicating that its overall chemical characteristics were more pronounced. The final PCA-based comprehensive evaluation model is as follows:

F1 = 0.176 × 1 − 0.092 × 2 + 0.210 × 3 + 0.244 × 4 + 0.175 × 5 + 0.221 × 6 + 0.199 × 7

F2 = 0.395 × 1 + 0.422 × 2 + 0.260 × 3 − 0.096 × 4 − 0.380 × 5 + 0.145 × 6 − 0.138 × 7

F = 0.694F1 + 0.306F2

### 3.5. Untargeted Metabolomic Profiling of PCA-Selected Representative SX Samples

**Figure 6:**
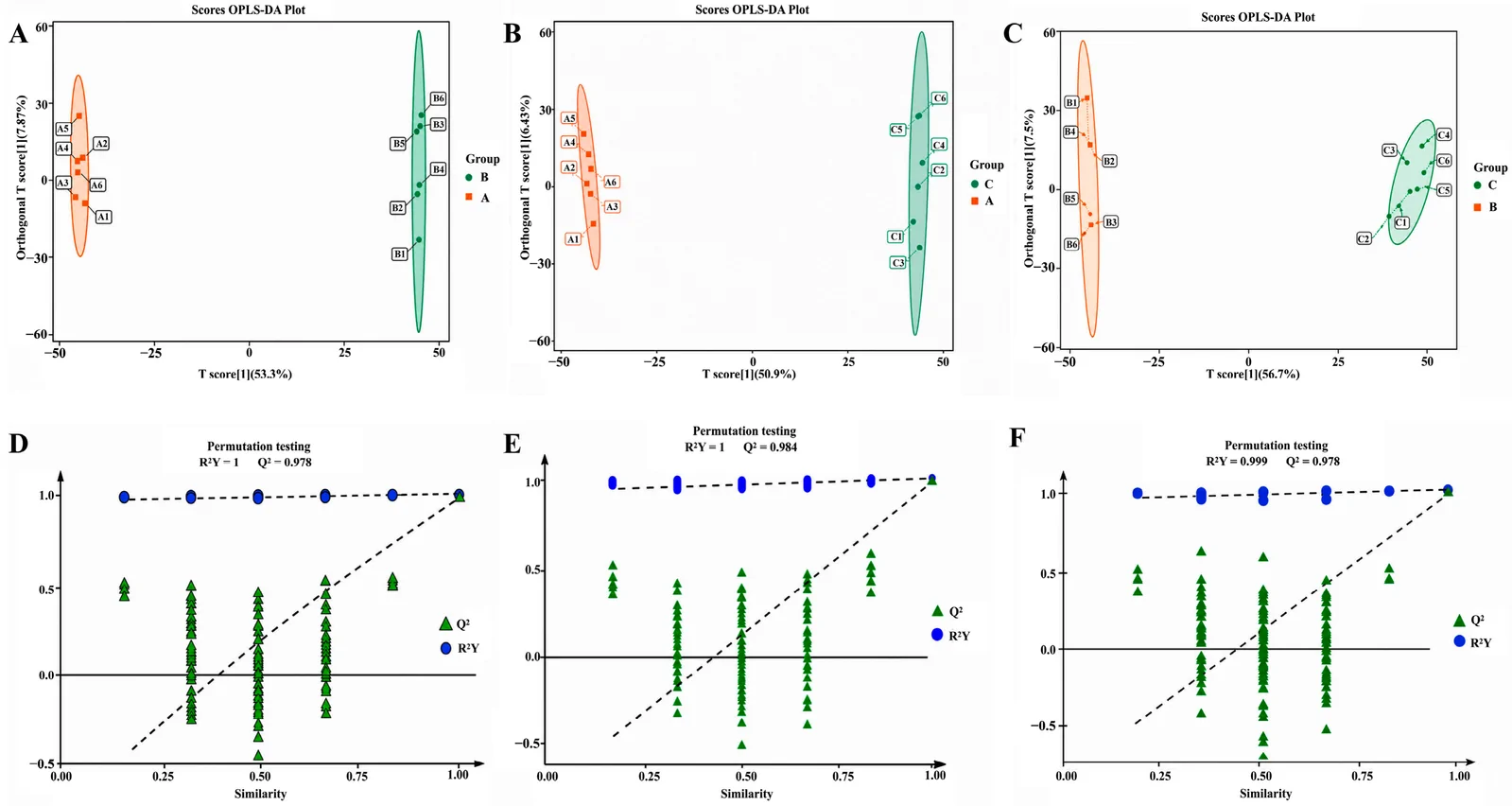
OPLS-DA score plots and permutation tests of metabolomic profiles among SX green tea infusions prepared by different extraction processes. (**A**–**C**) OPLS-DA score plots for the pairwise comparisons of SX-EAE-R58 vs. SX-HWE-R18, SX-HWE-R18 vs. SX-CB-R107, and SX-EAE-R58 vs. SX-CB-R107, respectively. (**D**–**F**) Corresponding permutation tests for the OPLS-DA models. In the metabolomics dataset, group A, group B, and group C correspond to SX-HWE-R18, SX-EAE-R58, and SX-CB-R107, respectively.

To characterize metabolomic differences among the three PCA-selected representative SX samples, pairwise OPLS-DA analyses were performed. Group A (SX-HWE-R18), group B (SX-EAE-R58), and group C (SX-CB-R107) corresponded to PCA ranks 18, 58, and 107, respectively. Because these samples differed in multiple extraction parameters, the observed metabolomic differences were interpreted as differences among the selected extraction-condition combinations rather than as isolated effects of HWE, EAE, or CB. As shown in Figure 6A–C, the three comparison groups (B vs. A, A vs. C, and B vs. C) were clearly separated in the score plots, with tight clustering within each group, indicating stable and reproducible metabolic differences. Permutation tests (Figure 6D–F) yielded R^2^Y values of 1.000, 1.000, and 0.999. For B vs. A, the R^2^Y and Q^2^ intercepts were 0.9615 and −0.7116, respectively; for A vs. C, 0.9402 and −0.7327; and for B vs. C, 0.9334 and −0.7969. In all three comparisons, R^2^Y was higher than Q^2^ and the Q^2^ regression-line intercept was negative. In all three models, R^2^Y was higher than Q^2^, and the Q^2^ regression-line intercept was below zero, supporting the stability of the models in permutation testing.

**Figure 7:**
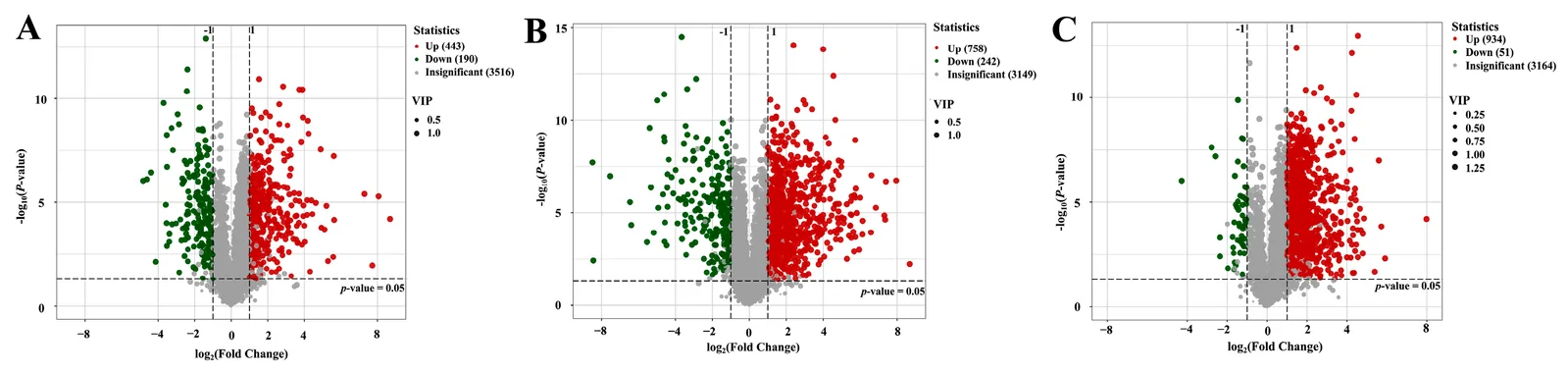
Volcano plots of differential metabolites among SX green tea infusions prepared by different extraction processes. (**A**) SX-EAE-R58 vs. SX-HWE-R18; (**B**) SX-HWE-R18 vs. SX-CB-R107; (**C**) SX-EAE-R58 vs. SX-CB-R107. Red, green, and gray dots represent upregulated, downregulated, and non-significant metabolites, respectively.

Differentially abundant metabolites (DAMs) were screened using the criteria of FC ≥ 2 or FC ≤ 0.5, VIP > 1, and *p* < 0.05, and visualized via volcano plots (Figure 7). In the SX-EAE-R58 vs. SX-HWE-R18 comparison, 633 DAMs were identified (443 upregulated, 190 downregulated). The number increased substantially in SX-HWE-R18 vs. SX-CB-R107 (1000 DAMs; 758 upregulated, 242 downregulated) and SX-EAE-R58 vs. SX-CB-R107 (985 DAMs; 934 upregulated, 51 downregulated). Under the applied screening criteria, more DAMs were detected in comparisons involving CB (SX-HWE-R18 vs. SX-CB-R107 and SX-EAE-R58 vs. SX-CB-R107) than in SX-EAE-R58 vs. SX-HWE-R18. This pattern suggests greater compositional differences in the comparisons involving CB.

**Figure 8:**
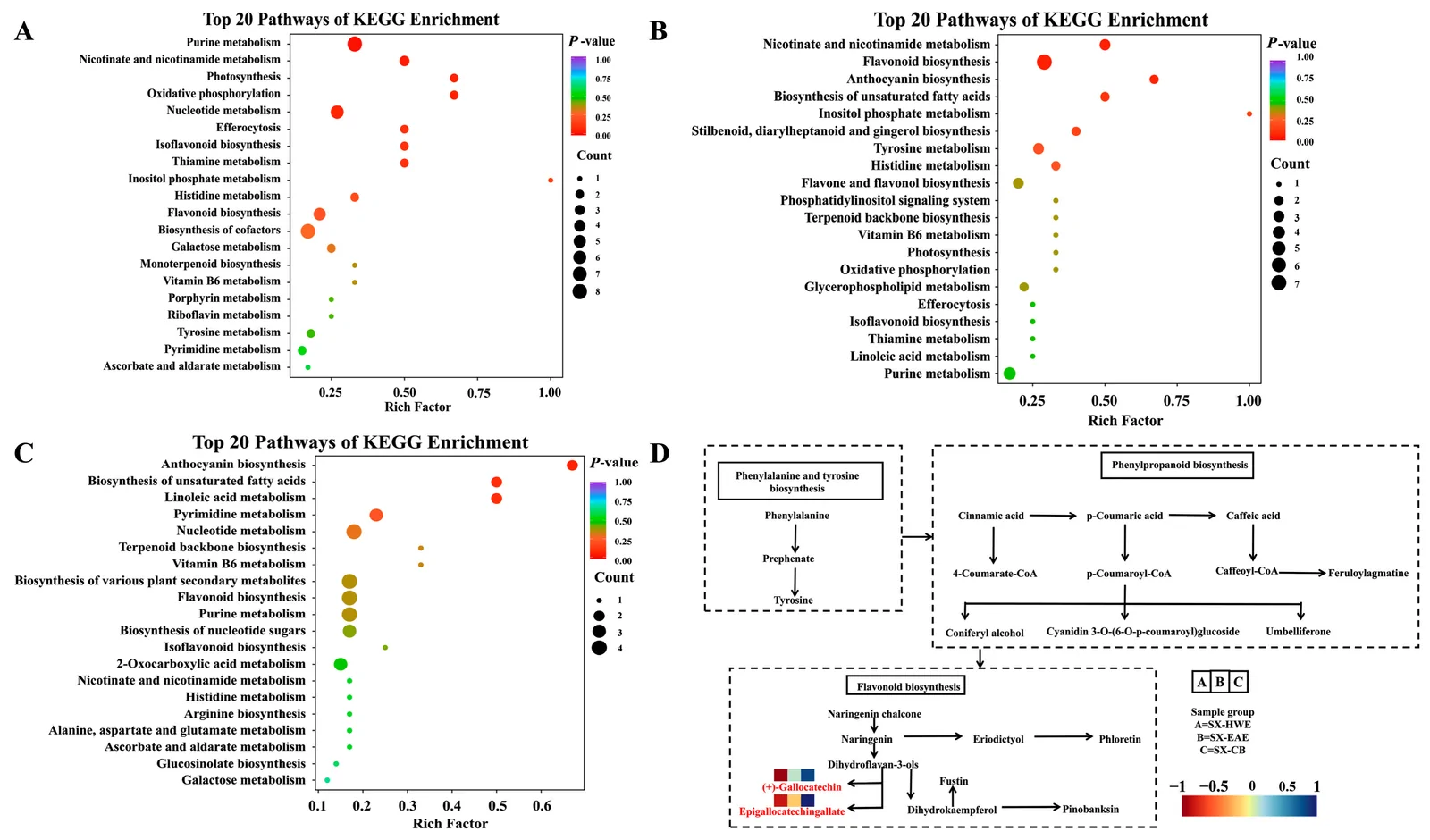
KEGG enrichment profiles and pathway-associated network of differentially abundant metabolites in SX green tea infusions. (**A**–**C**) Bubble plots show the 20 KEGG pathways with the lowest *p*-values for each pairwise comparison of differential metabolites for each pairwise comparison: SX-EAE-R58 vs. SX-HWE-R18 (**A**), SX-HWE-R18 vs. SX-CB-R107 (**B**), and SX-EAE-R58 vs. SX-CB-R107 (**C**). (**D**) KEGG metabolic network constructed based on pathway-associated differential metabolites. In the metabolomics dataset, group A, group B, and group C correspond to SX-HWE-R18, SX-EAE-R58, and SX-CB-R107, respectively.

KEGG enrichment analyses (Figure 8A–C) mapped the DAMs to pathways annotated as purine metabolism, nicotinate and nicotinamide metabolism, nucleotide metabolism, flavonoid biosynthesis, flavonol biosynthesis, anthocyanin biosynthesis, phenylpropanoid biosynthesis, and unsaturated fatty acid biosynthesis. In the SX-HWE-R18 vs. SX-CB-R107 and SX-EAE-R58 vs. SX-CB-R107 comparisons, metabolites associated with flavonoid- and plant secondary metabolite-related pathways were particularly prominent, reflecting distinct extraction and release patterns under CB conditions. The KEGG-based metabolic network (Figure 8D) further showed that several DAMs were associated with phenylalanine/tyrosine-, phenylpropanoid-, and flavonoid-related pathways. (+)-Gallocatechin and EGCG showed different relative abundance patterns among the three representative SX samples. These results indicate that metabolites associated with catechin- and flavonoid-related pathways contributed to the chemical differences among the selected samples. However, KEGG annotation does not demonstrate activation of these pathways or de novo biosynthesis during extraction.

## 4. Discussion

This study examined how three extraction methods affected water-soluble extracts and key chemical compounds in green tea infusions. The work used a stepwise analytical framework. Single-factor experiments were first used for preliminary screening. RSM was then applied for process optimization. HPLC was used to quantify selected target compounds. PCA was used to evaluate the overall chemical profile. Untargeted metabolomics was used to explore metabolic differences among representative samples.

The single-factor results are shown in Figure 1, Figures S1 and S2, whereas the RSM results are shown in Figure 2 and Figures S3–S5. These findings demonstrated that the release of soluble compounds was influenced by the solid-to-liquid ratio, extraction time, and particle size. This mechanism was primarily associated with extraction equilibrium, cell wall disruption, and solid-liquid mass transfer. The solvent’s ability to dissolve was enhanced by increasing solvent availability initially favored mass transfer, whereas extending extraction time allowed a larger fraction of soluble intracellular constituents to partition into the aqueous phase. The area of contact between the solvent and tea powder increased with decreasing particle size. The diffusion path was also shortened. These elements did not exhibit a straightforward beneficial impact. The extraction driving power may be diminished by an excessively high solid-to-liquid ratio. Extracting ester-type catechins for an extended period may encourage their breakdown or epimerization. The observed decrease in WEC at particle sizes finer than 60 mesh may reflect counteracting effects, including greater retention during filtration, aggregation of fine particles, or adsorption or association of soluble compounds with particulate material. Similar results were seen in recent research on green tea catechin extraction employing HWE [23], UAE [7], PEF [6], and MAE [24]. The necessity of enhancing mass transfer while preserving catechin stability was also emphasized by these investigations. WEC release was influenced by interactions between the three parameters rather than by a single element alone, as demonstrated by the response surface curves and contour plots.

The three extraction techniques had varying effects on WEC and the six quality-related chemicals, according to targeted HPLC data. HWE usually increased the release of WEC, caffeine, and most catechins. This impact might be related to quicker molecular diffusion and cell wall disintegration at high temperatures. Certain bound polyphenols and flavonoid derivatives may be released more readily when EAE is present. The enzymatic hydrolysis of pectin networks and cell wall polysaccharides is probably connected to this process. A softer extraction pattern was displayed by CB. Although the overall yield was decreased, the excessive release of bitter and astringent chemicals was lessened. Astringency and bitterness in tea infusions are primarily caused by catechins and caffeine [1]. Umami, aftertaste, and flavor modification are more closely associated with L-theanine and free amino acids. It should be emphasized that WEC was the sole response variable used in the present RSM optimization. Accordingly, the resulting conditions represent optima for the extraction of total water-extractable matter rather than for the overall chemical or sensory quality of the infusion. The subsequent HPLC analysis showed that the WEC-optimized conditions did not necessarily maximize individual catechins, caffeine, or L-theanine. This distinction is important because increases in total soluble extraction do not necessarily correspond to a more desirable chemical balance or sensory profile. A limitation of the present study is that multi-response optimization was not performed. Future studies could incorporate WEC together with total catechins, individual catechins, L-theanine, caffeine-related indices, and sensory responses into a multi-response optimization framework to identify conditions that better balance extraction efficiency and infusion characteristics. Because of this, higher WEC or EGCG content does not always translate into higher-quality infusions. EGCG, ECG, EC, EGC, caffeine, and L-theanine balance is also crucial. Because individual compounds may exhibit different extraction and degradation behaviors, future studies could employ multi-response RSM or compound-specific mathematical models to simultaneously optimize extraction yield and selected bioactive constituents. Such approaches may provide a more detailed description of compound-dependent extraction behavior [25]. Every extraction technique demonstrated a unique benefit. Although HWE increased release efficiency, it may also increase the risk of catechin epimerization and bitter chemicals. EAE may aid in the release of certain cell-wall-bound molecules, but it did not enhance all target chemicals. Although CB produced a smoother release profile, its total efficiency was lower. Low temperatures can retain more chlorophyll and decrease the flavonoid extraction ratio. This could result in a sweet taste, umami, floral scent, and a greener hue for the infusion [8]. The proportional amounts of catechins [26], amino acids, and alkaloids [27] can vary depending on the extraction intensity.

A more comprehensive understanding of the sample differences was provided by the PCA-based chemical analysis. The multi-indicator space did not have a random distribution of various tea samples and procedure combinations. Their positions were mainly shaped by WEC, catechins, caffeine, and L-theanine. The loading plot in Figure 5D showed different roles for these indicators. Catechins, L-theanine, and WEC contributed more to PC1. Caffeine mainly separated the samples along PC2. This pattern suggests that each indicator had a different weight in explaining the chemical profile of the tea infusions. This result is consistent with previous tea metabolomics studies. These studies reported that non-volatile tea quality is affected by catechins, amino acids, alkaloids, flavonoid glycosides, organic acids, and lipid derivatives [28]. A single indicator cannot fully describe tea liquor quality [29].

Untargeted metabolomics showed clear differences among the three selected SX samples. These differences were reflected in both the relative abundance and composition of detectable metabolites. The OPLS-DA score plots separated the three groups, and the volcano plots further showed differences in the abundance of numerous metabolites. Because the selected samples were prepared under different extraction-condition combinations, these differences should not be attributed to a single extraction factor. Instead, they may reflect the combined effects of extraction temperature, extraction time, solid-to-liquid ratio, particle size, enzymatic treatment, and other condition-dependent factors on the release, solubility, stability, and detectable abundance of pre-existing tea metabolites. The profiles of amino acids, flavonoids, catechins, alkaloids, and lipids can be altered by processing conditions, as reported in studies on Longjing green tea [12], pan-fired green tea [26], and teas subjected to different processing technologies [27]. Yu et al. investigated non-volatile metabolite alterations during round green tea processing using untargeted metabolomics. Their findings demonstrated that catechins, flavonoid glycosides, and amino acid derivatives were impacted by the degree of pan-firing [26]. Ni et al. investigated summer-autumn steaming green tea flakes using a combination of metabolomics and sensomics. They found that organic acids, theanine, and catechins were crucial for the development of taste [30]. The production of tea was the primary focus of these investigations. The infusion step is the focus of this investigation. Temperature, time, tissue disturbance, and enzymatic activity can all influence the migration and release of non-volatile metabolites into the final tea beverage.

KEGG enrichment and network analyses showed that several DAMs were annotated to phenylpropanoid-, flavonoid-, flavonol-, phenylalanine/tyrosine-, and purine-related pathways. In tea plants, phenylalanine serves as an upstream precursor of the phenylpropanoid pathway, which contributes to the formation of cinnamic acid, p-coumaric acid, caffeic acid, and downstream flavonoid derivatives, including catechins and flavonols [31,32,33]. In the present infusion study, however, these pathway annotations should not be interpreted as evidence that these biosynthetic pathways were activated during extraction. Rather, the enrichment pattern indicates that pre-existing metabolites associated with these pathways contributed to the compositional differences among the selected tea infusions. Several DAMs, including naringenin chalcone, phloretin, pinobanksin, fustin, (+)-gallocatechin, and EGCG, were associated with the flavonoid-related network. Their different relative abundances may reflect differences in extraction, release, solubility, or stability under the investigated extraction conditions. EGCG is sensitive to extraction time [34], pH [35], and temperature [32], and catechin stability can also be influenced by oxygen availability, metal ions, and other processing factors [36]. Therefore, the observed differences in catechin-related compounds are more appropriately interpreted as extraction-associated compositional differences rather than evidence of de novo biosynthesis. High-temperature cell wall breakdown and quick polyphenol release in this study may be linked to the increased EGCG content during HWE. Some DAMs differed under the EAE condition; however, without an enzyme-free control at 50 °C, these differences cannot be attributed specifically to enzymatic action. The WEC validation was conducted only to verify the predictive performance of the BBD-RSM models under the WEC-optimized conditions and was independent of the metabolomics analysis, which involved three PCA-selected representative SX samples without an enzyme-free control. Although CB did not maximize yield, it produced a milder release profile and may reduce bitterness.

A further significance of this study lies in integrating single-factor analysis and RSM for extraction optimization, HPLC for targeted quantification, PCA for multivariate chemical characterization, and untargeted metabolomics for characterizing metabolite- and pathway-associated differences among representative samples. This technique coincides with the recent trend in tea research that stresses integrated analysis of quality indicators [29]–multivariate statistics [26]–metabolic networks [37]. But there are a few restrictions to be aware of. First, the results cannot be completely extended to all green tea kinds because untargeted metabolomics was only used on SX tea samples. Second, KEGG pathway analysis is annotation-based and cannot directly demonstrate enzymatic activity, biosynthetic flux, pathway activation, or de novo biosynthesis during extraction. Therefore, the KEGG results in this study should be interpreted as pathway-associated metabolite patterns rather than evidence of active metabolic processes occurring during tea infusion preparation. Third, the PCA composite score is purely chemical and lacks sensory correlation. Fourth, no multiple-testing correction was applied to the metabolomics *p*-values; therefore, the DAM screening results and the pathway patterns derived from these screened metabolites should be regarded as exploratory and require further validation. Future studies should integrate sensory evaluation, volatile compound profiling, antioxidant activity assays, and storage stability validation to establish a “processing–components–sensory–stability” coupled model more suitable for green tea beverage development.

## 5. Conclusions

This study examined three types of green tea (AH, SX, and YN) to compare the effects of three extraction methods (HWE, EAE, and CB) on the chemical composition of green tea infusions. We established an analytical workflow integrating WEC optimization, HPLC-based targeted quantification, PCA-based comprehensive chemical scoring, and homogeneous non-targeted metabolomics. Single-factor experiments and BBD-RSM analysis indicated that the solid-to-liquid ratio, extraction time, and tea particle size all influenced the WEC of the tea infusion. The WEC-optimized conditions varied among the tea sample–process combinations, and the absolute relative deviations between the predicted and experimentally measured WEC values ranged from 0.12% to 0.81%, with all deviations below 1.0%, indicating generally close agreement between the model predictions and experimental measurements. Under the optimized WEC conditions, HPLC analysis revealed that the contents of EGC, EC, EGCG, ECG, caffeine, and L-theanine exhibited significant differences across different tea sample-process combinations, indicating that the optimal WEC conditions do not necessarily correspond to the optimal content of any single targeted component. The PCA-derived composite chemical score was used to select three representative SX samples from the high-, medium-, and low-score regions. These samples corresponded to PCA ranks 18, 58, and 107 and represented specific experimental condition combinations rather than the RSM-optimized infusions. Untargeted metabolomics further showed distinct compositional profiles among the selected representative SX samples. The DAMs were predominantly annotated to KEGG pathways related to phenylalanine/tyrosine, phenylpropanoid, flavonoid, and purine metabolism. These differences are interpreted as reflecting differential release and stability of pathway-associated metabolites under different extraction conditions, rather than pathway activation or de novo biosynthesis during extraction. Overall, this study elucidated the effects of different extraction processes on the chemical characteristics of green tea infusions across WEC, targeted constituents, and metabolomic profiles, providing data reference for selecting extraction processes and evaluating the chemical quality of green tea infusions.

## Figures and Tables

**Table 1:** Principal Component Analysis: Loads and Scaling Factors.

Indicator	Loading PC1	Loading PC2	Coefficient PC1	Coefficient PC2
EGC	0.670	0.663	0.176	0.395
Caffeine	−0.349	0.709	−0.092	0.422
EC	0.802	0.436	0.210	0.260
EGCG	0.932	−0.161	0.244	−0.096
ECG	0.666	−0.638	0.175	−0.380
L-theanine	0.843	0.244	0.221	0.145
WEC	0.760	−0.232	0.199	−0.138

Note: The coefficients used in the exploratory composite-score calculation were obtained by dividing each PCA loading by the corresponding eigenvalue, following Zhang et al. [16]. These coefficients are specific to the referenced composite-score formulation and are not standard PCA score coefficients.

## Data Availability

The original contributions presented in the study are included in the article/Supplementary Materials further inquiries can be directed to the corresponding authors.

## Supplementary Materials

The following supporting information can be downloaded at https://www.mdpi.com/article/10.3390/foods15203661/s1, Figure S1: Single-factor optimization of extraction conditions for WEC in AH green tea infusions; Figure S2: Single-factor optimization of extraction conditions for WEC in YN green tea infusions; Figure S3: Two-dimensional contour plots showing the effects of extraction variables on WEC in SX green tea infusions; Figure S4: Response surface and contour plots of extraction variables on WEC in AH green tea infusions; Figure S5: Response surface and contour plots of extraction variables on WEC in YN green tea infusions; Table S1: Mapping of LC-MS/MS analytical files to PCA ranks, experimental sample codes, and extraction conditions; Table S2: Coded and actual factor levels used in the Box–Behnken design; Table S3: Complete ANOVA for the quadratic response surface models of WEC; Table S4: Model Fit Statistics for the Nine RSM Models of WEC; Table S5: Quadratic models and WEC-optimized conditions; Table S6: Validation of response surface models by comparing predicted and experimental water-extractable content values under optimized extraction conditions; Table S7: Water-soluble extract content in green tea infusion and HPLC-determined levels of target chemical constituents across all experimental nodes; Table S8: Methodological validation parameters for the HPLC quantitative analysis of six target compounds in green tea infusion; Table S9: Standardized values of seven chemical indicators; Table S10: Major Component Scores and Comprehensive Chemical Scores for All Samples. Table S11: Liquid phase gradient setting parameters; Table S12: Mass spectrum parameters; Table S13: Differential metabolites and identification results; Table S14: B vs. A sample data; Table S15: A vs. C sample data; Table S16: B vs. C sample data.
