# Supplementary material for: An Integrated Framework for Green Tea Infusion Chemical Characterization: Process Optimization, PCA-Based Chemometric Evaluation, and Untargeted Metabolomic Profiling
Source: Foods. 2026 Oct 15;15(20):3661. doi: 10.3390/foods15203661 (PMC13703727; doi:10.3390/foods15203661)
Supplement: Supplementary file 1 [file foods-15-03661-s001.zip › supplementary materials (clean version).pdf]

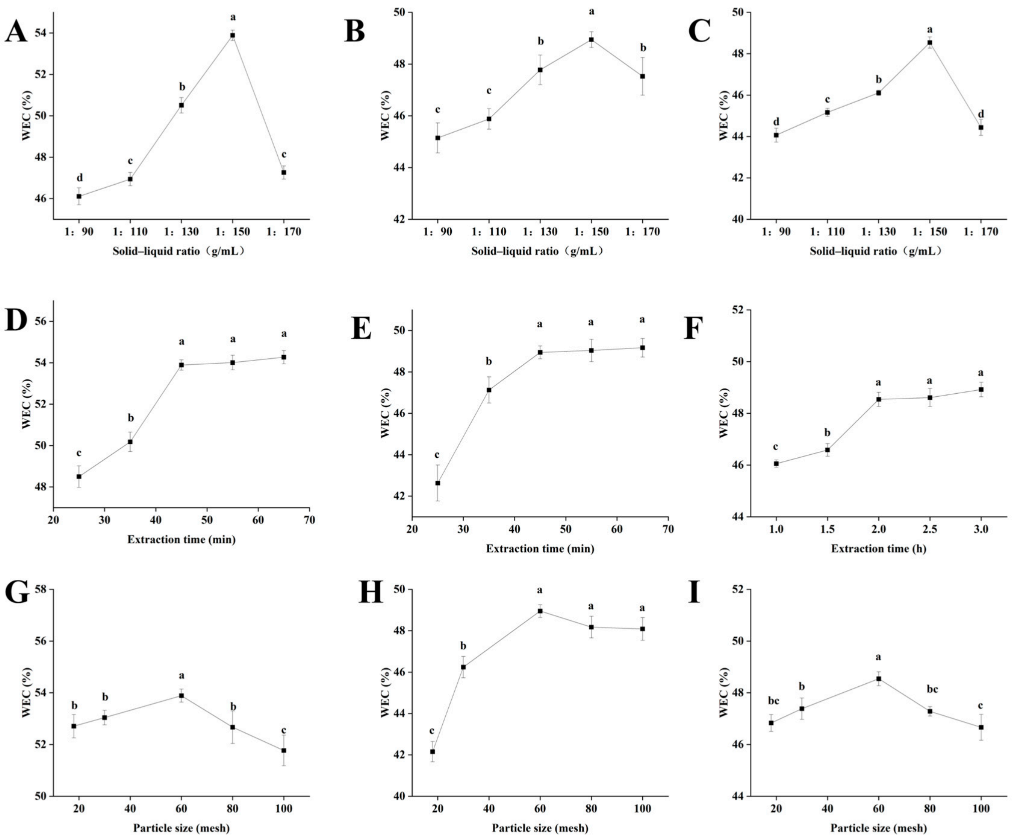

Figure S1. Single-factor optimization of extraction conditions for WEC in AH green tea infusions.(A-C) WEC at different solid-to-liquid ratios under HWE, EAE, and CB, respectively. (D-F) WEC at different extraction times under HWE, EAE, and CB, respectively. (G-I) WEC at different particle sizes under HWE, EAE, and CB, respectively. Different lowercase letters indicate significant differences among groups within the same factor ($p < 0.05$).

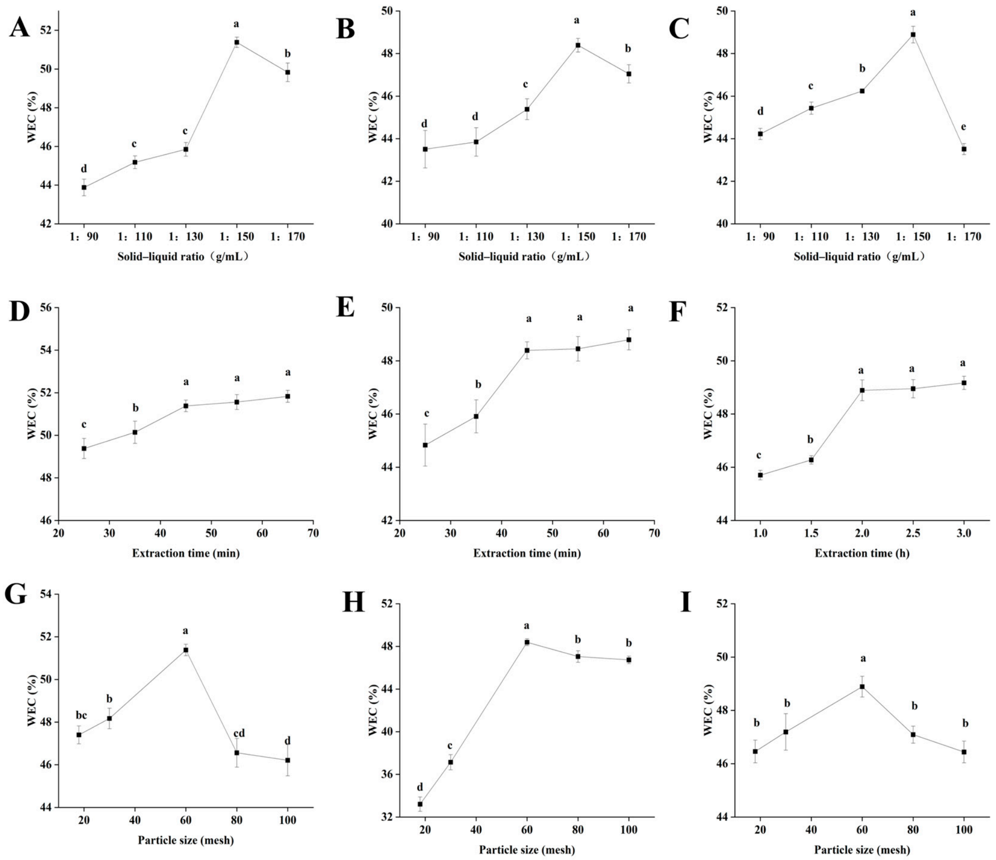

Figure S2. Single-factor optimization of extraction conditions for WEC in YN green tea infusions.(A-C) WEC at different solid-to-liquid ratios under HWE, EAE, and CB, respectively. (D-F) WEC at different extraction times under HWE, EAE, and CB, respectively. (G-I) WEC at different particle sizes under HWE, EAE, and CB, respectively. Different lowercase letters indicate significant differences among groups within the same factor ($p < 0.05$).

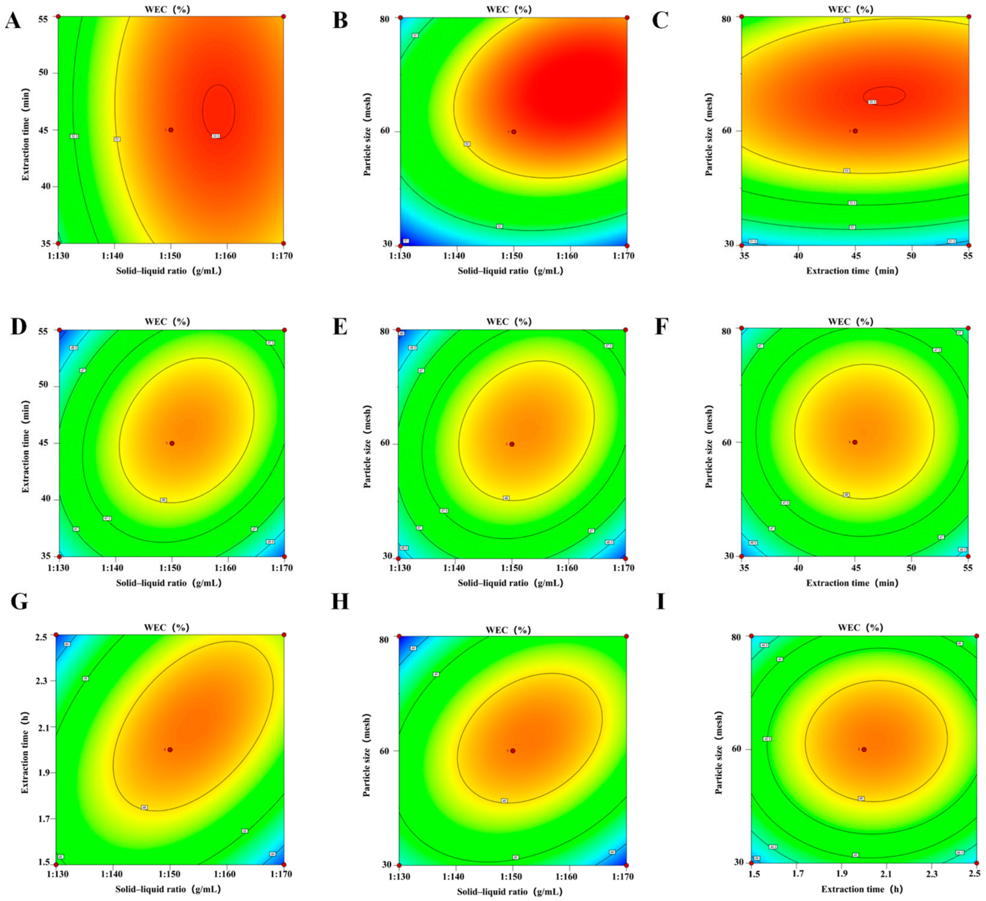

Figure S3. Two-dimensional contour plots showing the effects of extraction variables on WEC in SX green tea infusions. (A–C), (D–F), and (G–I) represent HWE, EAE, and CB, respectively. Within each extraction process, the plots show the interactions between solid-to-liquid ratio and extraction time, solid-to-liquid ratio and particle size, and extraction time and particle size, respectively.

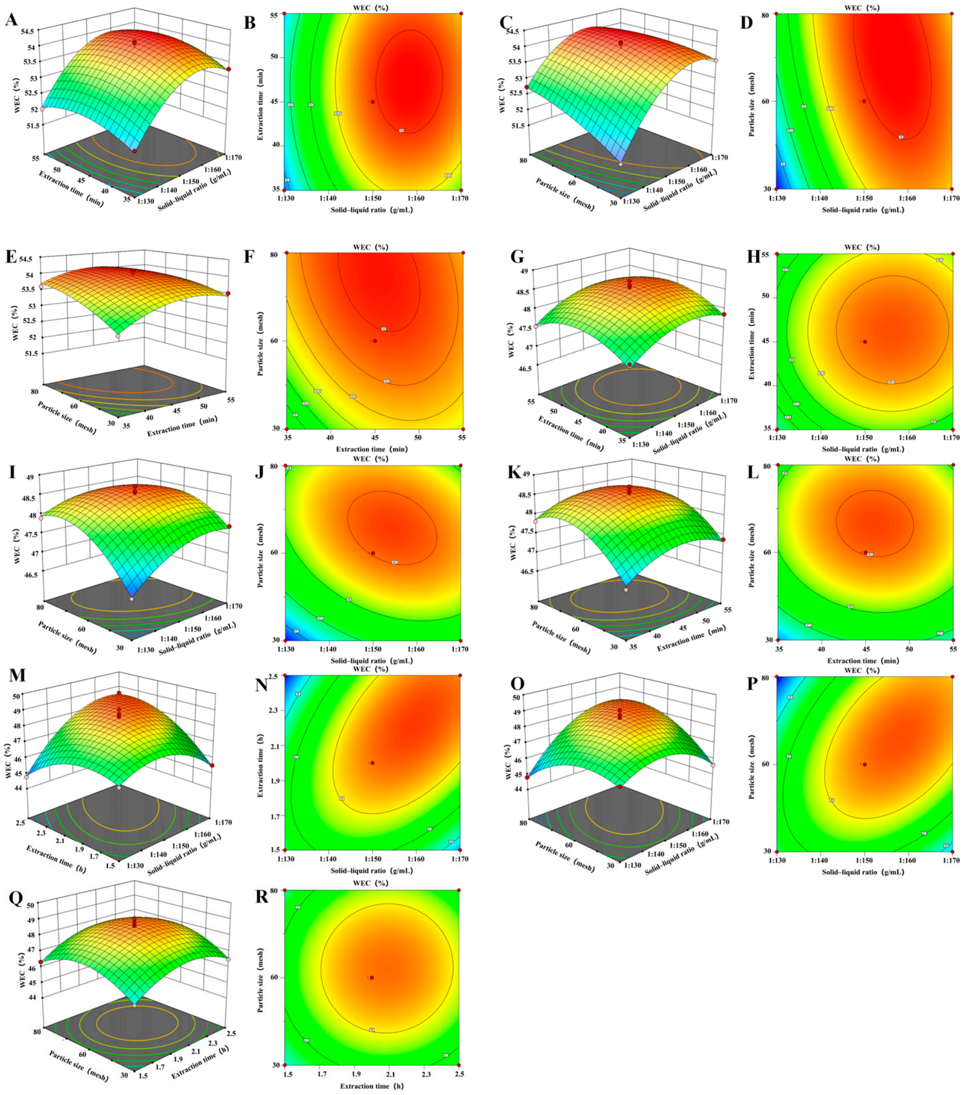

Figure S4. Response surface and contour plots of extraction variables on WEC in AH green tea infusions. (A,B), (G,H), and (M,N) show the interaction between solid-to-liquid ratio and extraction time at a fixed particle size under HWE, EAE, and CB, respectively. (C,D), (I,J), and (O,P) show the interaction between solid-to-liquid ratio and particle size at a fixed extraction time under HWE, EAE, and CB, respectively. (E,F), (K,L), and (Q,R) show the interaction between extraction time and particle size at a fixed solid-to-liquid ratio under HWE, EAE, and CB, respectively.

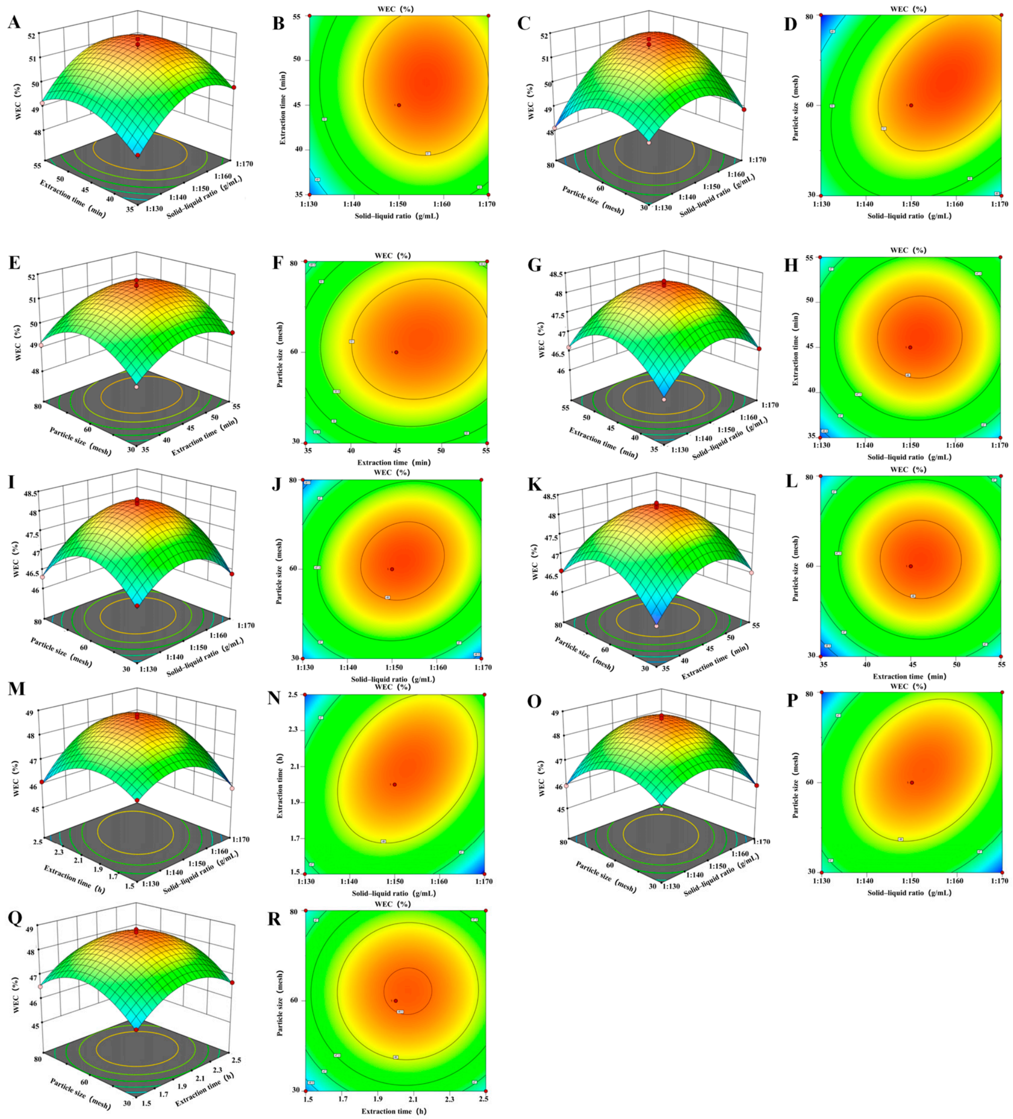

Figure S5. Response surface and contour plots of extraction variables on WEC in YN green tea infusions. (A,B), (G,H), and (M,N) show the interaction between solid-to-liquid ratio and extraction time at a fixed particle size under HWE, EAE, and CB, respectively. (C,D), (I,J), and (O,P) show the interaction between solid-to-liquid ratio and particle size at a fixed extraction time under HWE, EAE, and CB, respectively. (E,F), (K,L), and (Q,R) show the interaction between extraction time and particle size at a fixed solid-to-liquid ratio under HWE, EAE, and CB, respectively.

Table S1. Mapping of LC-MS/MS analytical files to PCA ranks, experimental sample codes, and extraction conditions.

LC-MS/MS files	Group	PCA rank	Experimental code	Extraction category	extraction conditions
A1–A6	A / SX-HWE-R18	18	S-F-S-M-4	HWE	95 ℃;1:150 (g:mL); 45 min; 80 mesh
B1–B6	B / SX-EAE-R58	58	S-M-S-L-1	EAE	50 ℃;1:90 (g:mL); 45 min; 60 mesh
C1–C6	C / SX-CB-R107	107	S-L-S-L-1	CB	20 ℃; 1:90 (g:mL); 2 h; 60 mesh
QC01–QC03	QC		Pooled QC		Equal aliquots of sample extracts were pooled

Note: R denotes PCA rank. HWE, hot-water extraction; EAE, enzyme-assisted extraction; CB, cold brewing; QC, pooled quality-control sample. SX-HWE-R18, SX-EAE-R58, and SX-CB-R107 represent the PCA-selected SX samples ranked 18, 58, and 107, respectively. These samples were freshly prepared according to the exact experimental conditions of the corresponding nodes and were not the RSM-optimized samples. QC samples were prepared by pooling equal aliquots of the sample extracts for monitoring analytical stability and reproducibility.

Table S2. Coded and actual factor levels used in the Box–Behnken design.

Extraction system	A (-1)	A (0)	A (+1)	A unit	B (-1)	B (0)	B (+1)	B unit	C (-1)	C (0)	C (+1)	C unit
HWE	1:130	1:150	1:170	g : ml	35	45	55	min	30	60	80	mesh
EAE	1:130	1:150	1:170	g : ml	35	45	55	min	30	60	80	mesh
CB	1:130	1:150	1:170	g : ml	1.5	2	2.5	h	30	60	80	mesh

Notes: A = solid-to-liquid ratio; B = extraction time; C = particle size.

Table S3. Complete ANOVA for the quadratic response surface models of WEC.

Combination	Source	SS	df	MS	F-value	*p*-value	Significance
AH-HWE	Model	10.05	9	1.12	53.08	< 0.0001	***
AH-HWE	A	4.30	1	4.30	204.35	< 0.0001	***
AH-HWE	B	0.1954	1	0.1954	9.29	0.0186	*
AH-HWE	C	0.6923	1	0.6923	32.92	0.0007	***
AH-HWE	AB	0.0053	1	0.0053	0.2541	0.6297	ns
AH-HWE	AC	0.3306	1	0.3306	15.72	0.0054	**
AH-HWE	BC	0.1023	1	0.1023	4.87	0.0632	ns
AH-HWE	A^2	3.39	1	3.39	161.06	< 0.0001	***
AH-HWE	B^2	0.5905	1	0.5905	28.08	0.0011	**
AH-HWE	C^2	0.1497	1	0.1497	7.12	0.0321	*
AH-HWE	Residual	0.1472	7	0.0210			
AH-HWE	Lack of Fit	0.0413	3	0.0138	0.5207	0.6906	ns
AH-HWE	Pure Error	0.1059	4	0.0265			
AH-HWE	Cor Total	10.19	16				
AH-EAE	Model	4.98	9	0.5534	20.68	0.0003	***
AH-EAE	A	0.4608	1	0.4608	17.22	0.0043	**
AH-EAE	B	0.0968	1	0.0968	3.62	0.0989	ns
AH-EAE	C	1.16	1	1.16	43.18	0.0003	***
AH-EAE	AB	0.0004	1	0.0004	0.0150	0.9061	ns
AH-EAE	AC	0.2401	1	0.2401	8.97	0.0201	*
AH-EAE	BC	0.0169	1	0.0169	0.6317	0.4528	ns
AH-EAE	A^2	0.6274	1	0.6274	23.45	0.0019	**
AH-EAE	B^2	0.7287	1	0.7287	27.24	0.0012	**
AH-EAE	C^2	1.35	1	1.35	50.42	0.0002	***
AH-EAE	Residual	0.1873	7	0.0268			
AH-EAE	Lack of Fit	0.0538	3	0.0179	0.5374	0.6814	ns
AH-EAE	Pure Error	0.1335	4	0.0334			

AH-EAE	Cor Total	5.17	16				
AH-CB	Model	31.90	9	3.54	37.37	< 0.0001	***
AH-CB	A	3.50	1	3.50	36.88	0.0005	***
AH-CB	B	0.9660	1	0.9660	10.18	0.0153	*
AH-CB	C	0.3916	1	0.3916	4.13	0.0817	ns
AH-CB	AB	5.45	1	5.45	57.48	0.0001	***
AH-CB	AC	4.41	1	4.41	46.49	0.0002	***
AH-CB	BC	0.0072	1	0.0072	0.0762	0.7905	ns
AH-CB	A^2	6.29	1	6.29	66.34	< 0.0001	***
AH-CB	B^2	4.30	1	4.30	45.28	0.0003	***
AH-CB	C^2	4.80	1	4.80	50.58	0.0002	***
AH-CB	Residual	0.6640	7	0.0949			
AH-CB	Lack of Fit	0.0486	3	0.0162	0.1054	0.9526	ns
AH-CB	Pure Error	0.6154	4	0.1538			
AH-CB	Cor Total	32.57	16				
SX-HWE	Model	13.84	9	1.54	46.12	< 0.0001	***
SX-HWE	A	2.14	1	2.14	64.24	< 0.0001	***
SX-HWE	B	0.0541	1	0.0541	1.62	0.2434	ns
SX-HWE	C	3.49	1	3.49	104.57	< 0.0001	***
SX-HWE	AB	0.0030	1	0.0030	0.0907	0.7720	ns
SX-HWE	AC	0.5700	1	0.5700	17.09	0.0044	**
SX-HWE	BC	0.0376	1	0.0376	1.13	0.3235	ns
SX-HWE	A^2	1.54	1	1.54	46.14	0.0003	***
SX-HWE	B^2	0.2019	1	0.2019	6.05	0.0434	*
SX-HWE	C^2	5.27	1	5.27	158.08	< 0.0001	***
SX-HWE	Residual	0.2335	7	0.0334			
SX-HWE	Lack of Fit	0.1041	3	0.0347	1.07	0.4545	ns
SX-HWE	Pure Error	0.1293	4	0.0323			
SX-HWE	Cor Total	14.08	16				

SX-EAE	Model	16.52	9	1.84	37.31	< 0.0001	***
SX-EAE	A	0.4019	1	0.4019	8.17	0.0244	*
SX-EAE	B	0.1880	1	0.1880	3.82	0.0914	ns
SX-EAE	C	0.2741	1	0.2741	5.57	0.0503	ns
SX-EAE	AB	0.8293	1	0.8293	16.86	0.0045	**
SX-EAE	AC	0.6916	1	0.6916	14.06	0.0072	**
SX-EAE	BC	0.0068	1	0.0068	0.1381	0.7212	ns
SX-EAE	A^2	4.72	1	4.72	96.01	< 0.0001	***
SX-EAE	B^2	3.63	1	3.63	73.89	< 0.0001	***
SX-EAE	C^2	4.29	1	4.29	87.16	< 0.0001	***
SX-EAE	Residual	0.3443	7	0.0492			
SX-EAE	Lack of Fit	0.0654	3	0.0218	0.3124	0.8168	ns
SX-EAE	Pure Error	0.2789	4	0.0697			
SX-EAE	Cor Total	16.86	16				
SX-CB	Model	23.03	9	2.56	62.62	< 0.0001	***
SX-CB	A	0.4180	1	0.4180	10.23	0.0151	*
SX-CB	B	0.3065	1	0.3065	7.50	0.0290	*
SX-CB	C	0.2671	1	0.2671	6.54	0.0377	*
SX-CB	AB	3.30	1	3.30	80.82	< 0.0001	***
SX-CB	AC	1.92	1	1.92	47.03	0.0002	***
SX-CB	BC	0.0312	1	0.0312	0.7624	0.4115	ns
SX-CB	A^2	4.39	1	4.39	107.42	< 0.0001	***
SX-CB	B^2	3.60	1	3.60	88.18	< 0.0001	***
SX-CB	C^2	7.07	1	7.07	173.02	< 0.0001	***
SX-CB	Residual	0.2860	7	0.0409			
SX-CB	Lack of Fit	0.0246	3	0.0082	0.1255	0.9402	ns
SX-CB	Pure Error	0.2614	4	0.0654			
SX-CB	Cor Total	23.31	16				
YN-HWE	Model	21.15	9	2.35	29.20	< 0.0001	***

YN-HWE	A	3.32	1	3.32	41.20	0.0004	***
YN-HWE	B	1.27	1	1.27	15.81	0.0054	**
YN-HWE	C	0.5304	1	0.5304	6.59	0.0371	*
YN-HWE	AB	0.0025	1	0.0025	0.0311	0.8651	ns
YN-HWE	AC	2.39	1	2.39	29.66	0.0010	***
YN-HWE	BC	0.1190	1	0.1190	1.48	0.2633	ns
YN-HWE	A^2	4.93	1	4.93	61.31	0.0001	***
YN-HWE	B^2	2.64	1	2.64	32.86	0.0007	***
YN-HWE	C^2	4.55	1	4.55	56.59	0.0001	***
YN-HWE	Residual	0.5633	7	0.0805			
YN-HWE	Lack of Fit	0.1009	3	0.0336	0.2910	0.8308	ns
YN-HWE	Pure Error	0.4624	4	0.1156			
YN-HWE	Cor Total	21.71	16				
YN-EAE	Model	9.56	9	1.06	53.25	< 0.0001	***
YN-EAE	A	0.1871	1	0.1871	9.38	0.0183	*
YN-EAE	B	0.2479	1	0.2479	12.43	0.0097	**
YN-EAE	C	0.1362	1	0.1362	6.83	0.0347	*
YN-EAE	AB	0.0052	1	0.0052	0.2585	0.6268	ns
YN-EAE	AC	0.1893	1	0.1893	9.49	0.0178	*
YN-EAE	BC	0.0018	1	0.0018	0.0880	0.7753	ns
YN-EAE	A^2	2.29	1	2.29	114.85	< 0.0001	***
YN-EAE	B^2	2.58	1	2.58	129.59	< 0.0001	***
YN-EAE	C^2	2.99	1	2.99	150.07	< 0.0001	***
YN-EAE	Residual	0.1396	7	0.0199			
YN-EAE	Lack of Fit	0.0283	3	0.0094	0.3388	0.7997	ns
YN-EAE	Pure Error	0.1113	4	0.0278			
YN-EAE	Cor Total	9.70	16				
YN-CB	Model	17.49	9	1.94	44.36	< 0.0001	***
YN-CB	A	0.2837	1	0.2837	6.47	0.0384	*

YN-CB	B	0.4758	1	0.4758	10.86	0.0132	*
YN-CB	C	0.3368	1	0.3368	7.69	0.0276	*
YN-CB	AB	1.54	1	1.54	35.09	0.0006	***
YN-CB	AC	1.17	1	1.17	26.62	0.0013	**
YN-CB	BC	0.0064	1	0.0064	0.1461	0.7137	ns
YN-CB	A^2	4.64	1	4.64	105.95	< 0.0001	***
YN-CB	B^2	3.56	1	3.56	81.34	< 0.0001	***
YN-CB	C^2	4.04	1	4.04	92.29	< 0.0001	***
YN-CB	Residual	0.3067	7	0.0438			
YN-CB	Lack of Fit	0.0700	3	0.0233	0.3945	0.7646	ns
YN-CB	Pure Error	0.2367	4	0.0592			
YN-CB	Cor Total	17.80	16				

Notes: SS = sum of squares; MS = mean square. Term-level SS values are partial sums of squares, with each term tested against the residual mean square of the full quadratic model. The 17-run BBD contains five center-point replicates, allowing pure error and lack-of-fit to be estimated. Predicted R^2 was calculated from PRESS. Adeq Precision was calculated as the range of fitted responses divided by the average prediction error.

Table S4. Model Fit Statistics for the Nine RSM Models of WEC.

**Combination**	**R^2**	**Adjusted R^2**	**Predicted R^2**	**C.V. (%)**	**Adeq Precision**	**Lack-of-fit *p*-value**
AH-HWE	0.9856	0.9670	0.9189	0.2722	21.5785	0.6906
AH-EAE	0.9638	0.9172	0.7931	0.3417	14.4838	0.6814
AH-CB	0.9796	0.9534	0.9466	0.6555	16.0020	0.9526
SX-HWE	0.9834	0.9621	0.8673	0.3480	18.0185	0.4545
SX-EAE	0.9796	0.9533	0.9121	0.4726	14.8324	0.8168
SX-CB	0.9877	0.9720	0.9656	0.4509	19.7103	0.9402
YN-HWE	0.9741	0.9407	0.8924	0.5673	15.0811	0.8308
YN-EAE	0.9856	0.9671	0.9354	0.3003	18.0411	0.7997
YN-CB	0.9828	0.9606	0.9163	0.4439	16.4792	0.7646

Note: R^2, coefficient of determination; Adjusted R^2, adjusted coefficient of determination; Predicted R^2, predicted coefficient of determination; C.V., coefficient of variation; Adeq Precision, adequate precision.

Table S5. Quadratic models and WEC-optimized conditions.

Combination	Quadratic regression equation (coded factors)	Optimal A (coded)	Optimal solid-to-liquid ratio (1:x, g:mL)	Optimal B (coded)	Optimal extraction time	Time unit	Optimal C (coded)	Optimal particle size (mesh)*	Predicted WEC (%)	R^2	Adjusted R^2
AH-HWE	$Y = 53.96 + 0.7330A + 0.1563B + 0.2942C + 0.0365AB - 0.2875AC - 0.1600BC - 0.8970A^2 - 0.3745B^2 - 0.1885C^2$	0.3356	156.71	0.1244	46.24	min	0.4715	69.43	54.16	0.9856	0.9670

AH-EAE	$Y = 48.51 + 0.2400A + 0.1100B + 0.3800C + 0.0100AB - 0.2450AC - 0.0650BC - 0.3860A^2 - 0.4160B^2 - 0.5660C^2$	0.2232	154.46	0.1129	46.13	min	0.2809	65.62	48.60	0.9638	0.9172
AH-CB	$Y = 48.54 + 0.6613A + 0.3475B + 0.2213C + 1.1675AB + 1.0500AC + 0.0425BC - 1.2225A^2 - 1.0100B^2 - 1.0675C^2$	0.7950	165.90	0.6422	2.32	h	0.5074	70.15	48.97	0.9796	0.9534
SX-HWE	$Y = 53.40 + 0.5175A + 0.0822B + 0.6603C - 0.0275AB + 0.3775AC + 0.0970BC - 0.6045A^2 - 0.2190B^2 - 1.1190C^2$	0.5467	160.93	0.2415	47.41	min	0.3977	67.95	53.68	0.9834	0.9621
SX-EAE	$Y = 48.34 + 0.2241A + 0.1533B + 0.1851C + 0.4553AB + 0.4158AC + 0.0412BC - 1.0590A^2 - 0.9290B^2 - 1.0090C^2$	0.1573	153.15	0.1239	46.24	min	0.1267	62.53	48.37	0.9796	0.9533
SX-CB	$Y = 46.35 + 0.2286A + 0.1957B + 0.1827C + 0.9086AB + 0.6931AC + 0.0882BC - 1.0210A^2 - 0.9250B^2 - 1.2957C^2$	0.2737	155.47	0.2475	2.12	h	0.1521	63.04	46.42	0.9877	0.9720
YN-HWE	$Y = 51.38 + 0.6438A + 0.3987B + 0.2575C + 0.0250AB + 0.7725AC + 0.1725BC - 1.0825A^2 - 0.7925B^2 - 1.0400C^2$	0.4075	158.15	0.2906	47.91	min	0.2992	65.98	51.61	0.9741	0.9407

YN-EAE	Y = 48.14 + 0.1529A + 0.1761B + 0.1305C + 0.0359AB + 0.2175AC − 0.0209BC − 0.7376A² − 0.7835B² − 0.8431C²	0.1199	152.40	0.1139	46.14	min	0.0914	61.83	48.17	0.9856	0.9671
YN-CB	Y = 48.54 + 0.1883A + 0.2439B + 0.2052C + 0.6200AB + 0.5400AC + 0.0400BC − 1.0500A² − 0.9200B² − 0.9800C²	0.1902	153.80	0.2001	2.10	h	0.1612	63.22	48.60	0.9828	0.9606

Notes: A = solid-to-liquid ratio; B = extraction time; C = particle size. All regression equations are expressed in coded variables.

Table S6. Validation of response surface models by comparing predicted and experimental water-extractable content values under optimized extraction conditions.

Category	AH-HWE	AH-EAE	AH-CB	SX-HWE	SX-EAE	SX-CB	YN-HWE	YN-EAE	YN-CB
Forecast Value(%)	54.15	48.60	48.97	53.67	48.37	46.42	51.61	48.17	48.60
Actual value(%)	53.71±0.41	48.53±0.37	48.68±0.39	53.48±0.33	48.1±0.32	46.61±0.35	51.55±0.42	48.07±0.26	48.35±0.36
Absolute relative deviation (%)	0.81	0.14	0.59	0.35	0.56	0.41	0.12	0.21	0.51

Note: Predicted values were obtained from the response surface models, whereas experimental values were determined under the corresponding WEC-optimized extraction conditions and are expressed as mean ± standard deviation (n = 3). Absolute relative deviation was calculated as ∣experimental value − predicted value∣ / predicted value × 100%.

Table S7. Water-soluble extract content in green tea infusion and HPLC-determined levels of target chemical constituents across all experimental nodes.

Sample code	EGC(mg/g)	Caffeine(mg/g)	EC(mg/g)	EGCG(mg/g)	ECG(mg/g)	L-theanine(mg/g)	WEC(%)
A-F-S-L-1	5.36 ± 0.22	28.19 ± 0.71	4.09 ± 0.29	48.53 ± 3.34	14.71 ± 1.32	22.65 ± 0.98	46.11 ± 0.41
A-F-S-L-2	5.78 ± 0.22	31.22 ± 0.47	4.13 ± 0.40	55.42 ± 4.23	17.31 ± 1.84	25.34 ± 1.27	46.94 ± 0.32
A-F-S-L-3	7.87 ± 0.43	39.05 ± 0.17	5.17 ± 0.43	73.43 ± 3.14	21.36 ± 1.44	28.59 ± 1.26	50.51 ± 0.37
A-F-S-L-4	8.57 ± 0.35	40.26 ± 0.22	5.50 ± 0.39	75.61 ± 3.25	22.77 ± 2.06	37.88 ± 1.61	53.89 ± 0.25
A-F-S-L-5	6.25 ± 0.22	37.80 ± 0.28	5.05 ± 0.34	65.67 ± 3.44	20.69 ± 1.55	26.58 ± 1.73	47.26 ± 0.32
A-F-S-T-1	4.01 ± 0.19	32.32 ± 0.32	4.77 ± 0.23	48.89 ± 1.78	16.34 ± 1.61	16.41 ± 0.53	48.50 ± 0.52
A-F-S-T-2	5.02 ± 0.26	32.55 ± 0.46	4.79 ± 0.31	55.51 ± 2.67	18.55 ± 1.76	21.65 ± 1.24	50.18 ± 0.47
A-F-S-T-3	8.57 ± 0.34	40.26 ± 0.27	5.50 ± 0.35	75.61 ± 3.01	22.77 ± 1.64	37.88 ± 2.44	53.89 ± 0.25
A-F-S-T-4	8.58 ± 0.37	40.33 ± 0.27	5.54 ± 0.32	75.91 ± 5.57	22.84 ± 2.23	37.96 ± 1.59	54.01 ± 0.35
A-F-S-T-5	8.71 ± 0.39	40.55 ± 0.26	5.63 ± 0.42	76.24 ± 5.00	22.94 ± 1.86	38.16 ± 2.13	54.27 ± 0.32
A-F-S-M-1	4.45 ± 0.29	25.76 ± 1.01	4.09 ± 0.38	46.76 ± 1.90	15.37 ± 1.12	15.17 ± 0.71	52.71 ± 0.45
A-F-S-M-2	6.87 ± 0.46	37.98 ± 0.25	8.99 ± 0.72	60.40 ± 4.05	19.94 ± 1.07	28.66 ± 1.53	53.04 ± 0.28
A-F-S-M-3	8.57 ± 0.58	40.26 ± 0.30	5.50 ± 0.43	75.61 ± 4.48	22.77 ± 1.58	37.88 ± 1.51	53.89 ± 0.25
A-F-S-M-4	5.75 ± 0.21	34.70 ± 0.38	6.83 ± 0.51	56.84 ± 2.99	18.98 ± 1.40	32.55 ± 1.26	52.67 ± 0.63
A-F-S-M-5	3.79 ± 0.26	32.69 ± 0.38	4.14 ± 0.38	54.28 ± 2.73	18.59 ± 1.22	16.37 ± 1.01	51.77 ± 0.59
A-F-R	8.47±0.34	40.06 ± 0.45	5.26±0.21	73.56±3.92	21.77±1.22	35.49±0.53	53.71 ± 0.41
A-L-S-L-1	4.45 ± 0.23	19.14 ± 1.19	3.50 ± 0.31	27.05 ± 1.47	7.58 ± 0.74	12.05 ± 0.90	44.07 ± 0.34
A-L-S-L-2	5.97 ± 0.25	20.57 ± 1.02	3.81 ± 0.22	29.49 ± 1.45	9.43 ± 1.09	12.36 ± 0.55	45.16 ± 0.19
A-L-S-L-3	6.90 ± 0.37	22.34 ± 1.16	4.12 ± 0.29	31.08 ± 1.99	10.34 ± 0.59	14.04 ± 0.77	46.11 ± 0.13
A-L-S-L-4	9.32 ± 0.50	28.08 ± 0.55	9.08 ± 0.75	46.21 ± 3.12	12.62 ± 0.90	31.93 ± 1.56	48.54 ± 0.27
A-L-S-L-5	5.49 ± 0.37	20.49 ± 0.82	3.64 ± 0.19	28.57 ± 1.44	8.45 ± 0.87	12.34 ± 0.56	44.44 ± 0.39
A-L-S-T-1	7.70 ± 0.36	22.15 ± 0.85	4.11 ± 0.26	36.07 ± 1.40	9.76 ± 0.85	15.01 ± 0.71	46.06 ± 0.15

A-L-S-T-2	7.77 ± 0.45	24.29 ± 1.00	4.12 ± 0.24	39.62 ± 1.52	10.24 ± 0.67	15.61 ± 0.63	46.58 ± 0.24
A-L-S-T-3	9.32 ± 0.51	28.08 ± 0.59	9.08 ± 0.84	46.21 ± 2.72	12.62 ± 1.06	31.93 ± 1.48	48.54 ± 0.27
A-L-S-T-4	9.32 ± 0.59	28.18 ± 0.72	9.08 ± 0.68	46.25 ± 2.20	12.62 ± 1.07	31.84 ± 1.49	48.61 ± 0.35
A-L-S-T-5	9.34 ± 0.73	28.30 ± 0.72	8.08 ± 0.51	46.27 ± 2.81	12.63 ± 0.66	32.04 ± 2.12	48.92 ± 0.28
A-L-S-M-1	7.10 ± 0.45	24.29 ± 0.72	4.25 ± 0.30	39.62 ± 3.02	11.47 ± 0.97	14.04 ± 0.71	46.83 ± 0.33
A-L-S-M-2	8.40 ± 0.56	26.48 ± 0.55	6.57 ± 0.45	41.86 ± 1.59	12.13 ± 0.94	23.39 ± 1.20	47.39 ± 0.41
A-L-S-M-3	9.32 ± 0.36	28.08 ± 0.55	9.08 ± 0.54	46.21 ± 1.95	12.62 ± 1.09	31.93 ± 1.62	48.54 ± 0.27
A-L-S-M-4	8.36 ± 0.53	24.32 ± 0.48	4.35 ± 0.41	39.64 ± 1.77	12.09 ± 0.86	14.52 ± 0.75	47.28 ± 0.18
A-L-S-M-5	6.59 ± 0.26	19.96 ± 0.77	3.78 ± 0.20	33.34 ± 2.12	9.35 ± 1.10	12.69 ± 0.76	46.66 ± 0.50
A-L-R	9.22±0.41	27.67 ± 0.72	8.86±0.56	45.52±3.04	12.27±0.89	30.74±0.79	48.68 ± 0.33
A-M-S-L-1	7.58 ± 0.30	26.64 ± 0.50	4.48 ± 0.23	41.35 ± 2.10	11.54 ± 1.21	15.06 ± 0.81	45.15 ± 0.58
A-M-S-L-2	8.52 ± 0.47	27.11 ± 0.79	4.72 ± 0.27	44.47 ± 1.73	11.63 ± 0.91	17.42 ± 1.10	45.88 ± 0.40
A-M-S-L-3	9.06 ± 0.35	30.61 ± 0.68	5.00 ± 0.26	47.22 ± 3.16	15.28 ± 1.26	18.00 ± 1.00	47.78 ± 0.57
A-M-S-L-4	9.91 ± 0.63	31.48 ± 0.67	6.88 ± 0.36	53.63 ± 3.64	15.93 ± 1.49	42.18 ± 1.59	48.95 ± 0.31
A-M-S-L-5	8.89 ± 0.52	28.82 ± 0.80	4.92 ± 0.41	45.81 ± 3.04	14.56 ± 1.70	17.85 ± 0.94	47.53 ± 0.73
A-M-S-T-1	6.19 ± 0.28	21.65 ± 1.06	4.59 ± 0.30	34.27 ± 1.97	9.29 ± 0.78	27.25 ± 1.41	42.63 ± 0.87
A-M-S-T-2	9.06 ± 0.40	27.57 ± 0.54	7.23 ± 0.47	48.51 ± 1.82	13.36 ± 0.89	37.18 ± 1.80	47.13 ± 0.63
A-M-S-T-3	9.91 ± 0.48	31.48 ± 0.43	6.88 ± 0.41	53.63 ± 2.85	15.93 ± 0.94	42.18 ± 2.91	48.95 ± 0.31
A-M-S-T-4	9.97 ± 0.59	31.52 ± 0.61	6.92 ± 0.45	53.73 ± 2.81	15.98 ± 1.38	42.27 ± 2.17	49.04 ± 0.54
A-M-S-T-5	10.13 ± 0.48	31.75 ± 0.56	6.98 ± 0.41	53.89 ± 4.07	16.11 ± 1.26	42.42 ± 2.56	49.17 ± 0.45
A-M-S-M-1	6.12 ± 0.20	26.16 ± 0.99	4.45 ± 0.30	44.47 ± 2.91	11.54 ± 0.96	13.62 ± 0.68	42.15 ± 0.48
A-M-S-M-2	8.39 ± 0.52	26.64 ± 0.68	4.48 ± 0.23	48.39 ± 2.62	11.91 ± 0.93	15.85 ± 0.70	46.24 ± 0.51
A-M-S-M-3	9.91 ± 0.61	31.48 ± 0.50	6.88 ± 0.44	53.63 ± 3.19	15.93 ± 1.12	42.18 ± 3.11	48.95 ± 0.31
A-M-S-M-4	9.06 ± 0.42	30.90 ± 0.73	5.00 ± 0.30	53.63 ± 3.88	14.61 ± 1.29	42.15 ± 2.70	48.17 ± 0.52
A-M-S-M-5	8.89 ± 0.42	30.61 ± 0.54	4.72 ± 0.32	53.07 ± 3.02	14.56 ± 1.43	42.00 ± 2.46	48.09 ± 0.55

A-M-R	9.69±0.48	31.03 ± 0.50	6.39±0.36	53.23±3.87	15.72±1.37	42.08±1.85	48.53±0.37
S-F-S-L-1	7.42 ± 0.53	32.93 ± 0.44	6.54 ± 0.56	56.16 ± 3.29	21.50 ± 1.95	22.42 ± 1.26	44.23 ± 0.29
S-F-S-L-2	7.51 ± 0.37	33.72 ± 0.39	6.64 ± 0.57	58.58 ± 3.96	23.29 ± 2.07	24.78 ± 1.09	44.81 ± 0.32
S-F-S-L-3	7.86 ± 0.39	37.51 ± 0.24	7.02 ± 0.58	69.66 ± 3.53	26.71 ± 2.29	25.45 ± 1.65	46.32 ± 0.45
S-F-S-L-4	9.33 ± 0.41	40.46 ± 0.16	7.77 ± 0.68	73.11 ± 3.92	28.68 ± 3.28	42.19 ± 2.78	53.18 ± 0.35
S-F-S-L-5	8.84 ± 0.46	40.30 ± 0.27	7.12 ± 0.49	70.36 ± 3.83	27.83 ± 2.37	29.42 ± 1.64	48.00 ± 0.43
S-F-S-T-1	6.72 ± 0.33	34.68 ± 0.29	6.18 ± 0.54	55.68 ± 2.27	24.30 ± 2.12	24.28 ± 1.09	48.99 ± 0.63
S-F-S-T-2	7.65 ± 0.58	37.42 ± 0.31	6.72 ± 0.40	63.57 ± 2.84	24.48 ± 1.90	28.53 ± 2.09	49.70 ± 0.55
S-F-S-T-3	9.33 ± 0.52	40.46 ± 0.22	7.77 ± 0.35	73.11 ± 4.95	28.68 ± 3.03	42.19 ± 1.90	53.18 ± 0.35
S-F-S-T-4	9.34 ± 0.60	40.59 ± 0.28	7.78 ± 0.55	73.24 ± 4.41	28.69 ± 2.12	42.31 ± 1.70	53.31 ± 0.29
S-F-S-T-5	9.39 ± 0.66	40.89 ± 0.16	7.86 ± 0.63	73.49 ± 3.94	28.96 ± 1.91	42.88 ± 2.51	53.59 ± 0.34
S-F-S-M-1	6.08 ± 0.36	32.44 ± 0.53	6.49 ± 0.58	56.44 ± 3.22	22.47 ± 2.13	19.02 ± 0.90	46.79 ± 0.56
S-F-S-M-2	7.25 ± 0.39	34.99 ± 0.33	7.12 ± 0.48	59.65 ± 4.05	23.65 ± 2.40	21.76 ± 1.43	48.81 ± 0.51
S-F-S-M-3	9.33 ± 0.52	40.46 ± 0.30	7.77 ± 0.52	73.11 ± 5.28	28.68 ± 2.52	42.19 ± 2.55	53.18 ± 0.35
S-F-S-M-4	7.37 ± 0.54	38.42 ± 0.21	7.22 ± 0.54	63.01 ± 2.75	25.95 ± 1.55	38.54 ± 1.87	49.17 ± 0.71
S-F-S-M-5	6.22 ± 0.34	34.57 ± 0.54	6.49 ± 0.45	57.70 ± 2.85	23.55 ± 1.92	19.40 ± 1.16	48.58 ± 0.64
S-F-R	9.53±0.50	40.85 ± 0.64	7.9±0.48	74.51±3.42	29.68±2.36	42.29±1.05	53.48 ± 0.33
S-L-S-L-1	3.22 ± 0.14	17.10 ± 1.25	3.56 ± 0.27	21.35 ± 1.23	8.65 ± 0.88	11.54 ± 0.66	43.20 ± 0.34
S-L-S-L-2	4.72 ± 0.24	19.07 ± 0.93	4.51 ± 0.28	24.52 ± 1.02	9.90 ± 1.04	15.09 ± 0.78	44.07 ± 0.24
S-L-S-L-3	5.43 ± 0.23	22.26 ± 0.81	4.54 ± 0.38	27.19 ± 2.08	9.97 ± 1.00	15.33 ± 0.78	44.57 ± 0.28
S-L-S-L-4	6.53 ± 0.38	31.46 ± 0.71	4.80 ± 0.40	30.21 ± 1.46	15.32 ± 1.61	15.46 ± 0.88	46.94 ± 0.41
S-L-S-L-5	4.07 ± 0.20	18.20 ± 0.87	4.51 ± 0.24	24.39 ± 0.83	9.40 ± 0.71	14.61 ± 1.05	43.33 ± 0.32
S-L-S-T-1	4.92 ± 0.30	19.76 ± 0.75	3.89 ± 0.37	26.35 ± 1.31	8.65 ± 0.78	11.40 ± 0.78	43.69 ± 0.12
S-L-S-T-2	5.20 ± 0.32	20.04 ± 1.07	4.34 ± 0.26	27.64 ± 1.24	9.24 ± 0.73	14.57 ± 0.76	43.98 ± 0.14
S-L-S-T-3	6.53 ± 0.30	31.46 ± 0.62	4.80 ± 0.27	30.21 ± 1.93	15.32 ± 1.21	15.46 ± 0.86	46.94 ± 0.41

S-L-S-T-4	6.54 ± 0.39	31.54 ± 0.36	4.82 ± 0.30	30.27 ± 1.53	15.32 ± 1.32	15.46 ± 0.83	47.02 ± 0.27
S-L-S-T-5	6.56 ± 0.31	31.61 ± 0.47	4.85 ± 0.23	30.31 ± 1.60	15.38 ± 1.28	15.50 ± 0.55	47.27 ± 0.33
S-L-S-M-1	4.22 ± 0.31	19.10 ± 1.38	3.95 ± 0.32	21.35 ± 1.20	8.65 ± 0.93	12.61 ± 0.77	44.95 ± 0.50
S-L-S-M-2	5.40 ± 0.28	25.68 ± 0.74	4.32 ± 0.42	27.96 ± 1.19	12.91 ± 1.08	13.21 ± 0.55	45.45 ± 0.33
S-L-S-M-3	6.53 ± 0.45	31.46 ± 0.67	4.80 ± 0.38	30.21 ± 1.28	15.32 ± 1.04	15.46 ± 0.54	46.94 ± 0.41
S-L-S-M-4	5.54 ± 0.34	26.40 ± 0.69	4.44 ± 0.33	28.36 ± 1.00	13.49 ± 1.32	13.83 ± 0.74	45.84 ± 0.26
S-L-S-M-5	5.37 ± 0.26	24.96 ± 0.63	4.25 ± 0.34	27.51 ± 1.40	12.73 ± 0.94	13.43 ± 0.61	45.26 ± 0.46
S-L-R	6.12±0.29	30.86 ± 0.87	4.65±0.31	29.90±1.57	15.13±1.06	15.21±0.71	46.61 ± 0.35
S-M-S-L-1	7.58 ± 0.50	26.55 ± 0.76	6.06 ± 0.36	38.58 ± 1.71	12.42 ± 1.20	9.49 ± 0.81	42.96 ± 0.65
S-M-S-L-2	7.69 ± 0.42	27.41 ± 0.51	6.07 ± 0.49	41.19 ± 2.00	13.18 ± 1.04	12.58 ± 0.57	43.71 ± 0.51
S-M-S-L-3	8.65 ± 0.54	27.79 ± 0.56	6.14 ± 0.55	45.94 ± 2.12	15.90 ± 1.29	14.19 ± 0.85	46.30 ± 0.49
S-M-S-L-4	10.43 ± 0.70	33.09 ± 0.59	7.27 ± 0.43	55.08 ± 2.70	18.96 ± 1.55	21.96 ± 0.96	48.58 ± 0.34
S-M-S-L-5	9.63 ± 0.60	31.55 ± 0.53	7.04 ± 0.62	51.27 ± 3.35	18.05 ± 1.56	17.38 ± 0.88	46.75 ± 0.45
S-M-S-T-1	8.11 ± 0.49	28.36 ± 0.48	6.05 ± 0.36	47.01 ± 3.30	16.66 ± 1.29	17.38 ± 1.25	46.01 ± 0.76
S-M-S-T-2	8.72 ± 0.33	29.77 ± 0.67	6.56 ± 0.35	48.40 ± 2.34	16.99 ± 1.49	21.38 ± 1.06	47.03 ± 0.53
S-M-S-T-3	10.43 ± 0.53	33.09 ± 0.30	7.27 ± 0.47	55.08 ± 3.91	18.96 ± 1.48	21.96 ± 1.21	48.58 ± 0.34
S-M-S-T-4	10.48 ± 0.60	33.24 ± 0.54	7.29 ± 0.43	55.14 ± 2.48	19.04 ± 1.52	22.09 ± 1.07	48.69 ± 0.49
S-M-S-T-5	10.52 ± 0.79	33.42 ± 0.50	7.32 ± 0.45	55.28 ± 2.49	19.18 ± 1.41	22.21 ± 1.14	48.82 ± 0.43
S-M-S-M-1	6.19 ± 0.30	26.55 ± 0.48	4.74 ± 0.44	38.58 ± 1.41	12.42 ± 0.98	9.49 ± 0.61	41.52 ± 0.51
S-M-S-M-2	7.58 ± 0.50	27.41 ± 1.03	6.06 ± 0.34	41.19 ± 3.00	13.18 ± 0.83	12.58 ± 0.77	43.45 ± 0.44
S-M-S-M-3	10.43 ± 0.41	33.09 ± 0.44	7.27 ± 0.49	55.08 ± 2.57	18.96 ± 1.15	21.96 ± 0.81	48.58 ± 0.34
S-M-S-M-4	8.65 ± 0.55	33.00 ± 0.43	6.14 ± 0.32	51.03 ± 2.87	17.32 ± 1.42	18.87 ± 1.16	48.14 ± 0.26
S-M-S-M-5	7.69 ± 0.31	27.79 ± 0.81	6.07 ± 0.32	45.94 ± 2.38	15.90 ± 1.01	17.38 ± 0.81	47.99 ± 0.27
S-M-R	10.13±0.47	32.59 ± 0.74	7.17±0.29	52.08±2.97	18.46±1.27	20.50±0.66	48.10 ± 0.32
Y-F-S-L-1	1.45 ± 0.11	30.21 ± 0.43	3.46 ± 0.23	37.38 ± 1.51	18.21 ± 1.27	12.14 ± 0.70	43.88 ± 0.43

Y-F-S-L-2	1.54 ± 0.14	32.35 ± 0.66	3.62 ± 0.22	40.98 ± 2.26	20.11 ± 2.01	13.10 ± 0.76	45.18 ± 0.33
Y-F-S-L-3	1.74 ± 0.17	39.00 ± 0.21	3.97 ± 0.26	48.73 ± 1.96	24.52 ± 2.35	13.20 ± 0.78	45.85 ± 0.35
Y-F-S-L-4	2.23 ± 0.20	44.37 ± 0.03	4.26 ± 0.20	54.51 ± 2.44	27.50 ± 2.75	15.24 ± 0.77	51.38 ± 0.27
Y-F-S-L-5	2.12 ± 0.23	42.49 ± 0.11	4.06 ± 0.29	54.14 ± 2.88	27.27 ± 2.70	13.39 ± 0.78	49.83 ± 0.48
Y-F-S-T-1	1.68 ± 0.14	38.26 ± 0.17	3.93 ± 0.30	50.10 ± 2.13	25.00 ± 1.38	10.00 ± 0.61	49.38 ± 0.47
Y-F-S-T-2	1.69 ± 0.15	40.35 ± 0.19	4.04 ± 0.22	52.64 ± 3.25	26.38 ± 2.65	13.10 ± 0.91	50.14 ± 0.52
Y-F-S-T-3	2.23 ± 0.21	44.37 ± 0.03	4.26 ± 0.29	54.51 ± 2.10	27.50 ± 1.59	15.24 ± 0.73	51.38 ± 0.27
Y-F-S-T-4	2.26 ± 0.18	44.56 ± 0.01	4.28 ± 0.28	54.67 ± 2.40	27.53 ± 2.01	15.30 ± 0.69	51.56 ± 0.35
Y-F-S-T-5	2.29 ± 0.23	44.72 ± 0.00	4.30 ± 0.25	54.93 ± 2.32	27.74 ± 1.95	15.48 ± 0.59	51.83 ± 0.28
Y-F-S-M-1	1.63 ± 0.17	39.88 ± 0.22	3.26 ± 0.28	48.23 ± 1.82	25.51 ± 2.17	13.10 ± 0.69	47.40 ± 0.42
Y-F-S-M-2	2.12 ± 0.17	40.61 ± 0.27	3.97 ± 0.27	48.50 ± 1.73	25.92 ± 1.81	14.94 ± 1.00	48.17 ± 0.48
Y-F-S-M-3	2.23 ± 0.21	44.37 ± 0.02	4.26 ± 0.33	54.51 ± 2.81	27.50 ± 2.17	15.24 ± 0.79	51.38 ± 0.27
Y-F-S-M-4	0.68 ± 0.08	39.44 ± 0.20	3.03 ± 0.25	47.96 ± 2.98	23.56 ± 1.56	12.75 ± 0.74	46.56 ± 0.67
Y-F-S-M-5	0.38 ± 0.04	37.92 ± 0.23	2.81 ± 0.26	47.93 ± 2.50	22.95 ± 1.63	12.55 ± 0.87	46.21 ± 0.73
Y-F-R	2.39±0.22	44.32 ± 0.67	4.41±0.33	55.28±2.27	28.04±1.48	15.24±0.62	51.55 ± 0.42
Y-L-S-L-1	0.71 ± 0.08	26.87 ± 0.58	2.79 ± 0.22	23.41 ± 1.46	12.17 ± 1.28	10.84 ± 0.44	44.22 ± 0.27
Y-L-S-L-2	1.06 ± 0.09	27.47 ± 0.62	2.85 ± 0.30	24.25 ± 1.70	12.43 ± 1.10	11.10 ± 0.65	45.43 ± 0.28
Y-L-S-L-3	1.57 ± 0.13	28.23 ± 0.64	3.01 ± 0.15	25.26 ± 1.43	12.91 ± 0.92	11.26 ± 0.54	46.24 ± 0.10
Y-L-S-L-4	1.86 ± 0.20	32.15 ± 0.50	3.22 ± 0.25	28.28 ± 1.07	15.23 ± 1.12	12.46 ± 0.55	48.89 ± 0.39
Y-L-S-L-5	0.68 ± 0.07	24.33 ± 1.04	2.56 ± 0.25	21.48 ± 0.89	10.53 ± 0.57	9.52 ± 0.52	43.51 ± 0.26
Y-L-S-T-1	0.46 ± 0.05	29.47 ± 0.50	2.93 ± 0.26	24.76 ± 1.49	13.23 ± 1.01	11.16 ± 0.65	45.70 ± 0.18
Y-L-S-T-2	0.58 ± 0.05	30.13 ± 0.55	3.03 ± 0.23	25.52 ± 1.11	14.45 ± 0.93	11.70 ± 0.56	46.28 ± 0.16
Y-L-S-T-3	1.86 ± 0.21	32.15 ± 0.71	3.22 ± 0.25	28.28 ± 1.43	15.23 ± 1.18	12.46 ± 0.66	48.89 ± 0.39
Y-L-S-T-4	1.86 ± 0.18	32.24 ± 0.48	3.23 ± 0.28	28.30 ± 1.86	15.31 ± 1.34	12.50 ± 0.60	48.95 ± 0.34
Y-L-S-T-5	1.87 ± 0.14	32.36 ± 0.51	3.25 ± 0.20	28.48 ± 1.06	15.45 ± 1.41	12.64 ± 0.63	49.17 ± 0.25

Y-L-S-M-1	0.76 ± 0.09	27.82 ± 0.40	2.70 ± 0.27	25.63 ± 1.43	14.18 ± 1.30	13.60 ± 0.75	46.46 ± 0.43
Y-L-S-M-2	1.07 ± 0.07	30.93 ± 0.67	3.05 ± 0.19	26.58 ± 1.10	14.72 ± 0.96	12.03 ± 0.68	47.19 ± 0.68
Y-L-S-M-3	1.86 ± 0.16	32.15 ± 0.52	3.22 ± 0.23	28.28 ± 1.57	15.23 ± 0.94	12.46 ± 0.86	48.89 ± 0.39
Y-L-S-M-4	0.94 ± 0.08	30.45 ± 0.60	3.01 ± 0.27	26.25 ± 1.01	14.71 ± 1.51	11.87 ± 0.65	47.09 ± 0.32
Y-L-S-M-5	0.59 ± 0.06	25.56 ± 0.87	2.57 ± 0.23	25.53 ± 1.60	14.20 ± 1.22	11.58 ± 0.58	46.44 ± 0.40
Y-L-R	1.82±0.11	32.11 ± 0.77	3.20±0.25	27.68±1.50	15.08±1.00	12.34±0.63	48.35 ± 0.36
Y-M-S-L-1	1.24 ± 0.13	27.90 ± 0.59	3.34 ± 0.26	27.87 ± 1.15	12.59 ± 0.87	16.16 ± 1.08	43.51 ± 0.88
Y-M-S-L-2	1.44 ± 0.10	31.18 ± 0.62	3.43 ± 0.28	32.11 ± 2.08	14.25 ± 1.23	16.86 ± 0.77	43.85 ± 0.67
Y-M-S-L-3	2.15 ± 0.18	36.87 ± 0.23	3.50 ± 0.23	38.56 ± 1.36	17.49 ± 1.49	17.40 ± 0.66	45.38 ± 0.49
Y-M-S-L-4	2.66 ± 0.23	39.58 ± 0.23	3.76 ± 0.26	44.34 ± 1.86	20.31 ± 1.44	20.76 ± 1.44	48.39 ± 0.32
Y-M-S-L-5	2.58 ± 0.26	37.24 ± 0.41	3.72 ± 0.23	42.35 ± 2.73	19.30 ± 1.42	19.51 ± 1.03	47.04 ± 0.43
Y-M-S-T-1	1.30 ± 0.10	37.01 ± 0.29	3.36 ± 0.22	36.15 ± 2.49	16.84 ± 1.67	12.10 ± 0.65	44.83 ± 0.79
Y-M-S-T-2	1.44 ± 0.14	37.52 ± 0.17	3.53 ± 0.28	39.96 ± 1.50	18.57 ± 1.58	16.86 ± 1.00	45.91 ± 0.62
Y-M-S-T-3	2.66 ± 0.24	39.58 ± 0.23	3.76 ± 0.19	44.34 ± 1.70	20.31 ± 2.01	20.76 ± 1.06	48.39 ± 0.32
Y-M-S-T-4	2.69 ± 0.20	39.84 ± 0.26	3.81 ± 0.30	44.41 ± 2.67	20.42 ± 1.39	20.82 ± 1.00	48.45 ± 0.46
Y-M-S-T-5	2.72 ± 0.21	40.21 ± 0.25	3.91 ± 0.30	44.54 ± 2.61	20.63 ± 2.37	20.96 ± 1.28	48.79 ± 0.38
Y-M-S-M-1	1.01 ± 0.10	26.04 ± 0.95	2.44 ± 0.19	28.32 ± 1.60	12.57 ± 1.01	10.15 ± 0.76	33.20 ± 0.67
Y-M-S-M-2	1.13 ± 0.12	29.60 ± 0.44	2.71 ± 0.28	33.56 ± 1.50	15.61 ± 0.99	12.58 ± 0.46	37.14 ± 0.71
Y-M-S-M-3	2.66 ± 0.28	39.58 ± 0.14	3.76 ± 0.27	44.34 ± 2.06	20.31 ± 1.61	20.76 ± 1.17	48.39 ± 0.32
Y-M-S-M-4	2.44 ± 0.19	37.34 ± 0.28	3.72 ± 0.30	42.24 ± 2.56	19.10 ± 1.56	17.86 ± 0.65	47.05 ± 0.54
Y-M-S-M-5	2.13 ± 0.21	35.94 ± 0.31	3.36 ± 0.18	41.52 ± 2.44	18.89 ± 1.67	15.98 ± 0.75	46.73 ± 0.36
Y-M-R	2.61±0.23	39.14 ± 0.78	3.64±0.24	44.08±2.66	19.67±1.28	20.28±0.46	48.07 ± 0.26

Note: In the extraction-process code, F, M, and L represent hot-water extraction, enzyme-assisted extraction, and cold brewing, respectively. In the experiment-type code, S and R represent single-factor experiments and optimized response surface conditions, respectively. For single-factor experiments, L, T, and M represent solid-to-liquid ratio, extraction time, and particle size, respectively; numbers 1–5 indicate the first to fifth levels of each single factor. Values are expressed as mean ± standard deviation.

Table S8. Methodological validation parameters for the HPLC quantitative analysis of six target compounds in green tea infusion.

Compound	Retention time (min)	Linear range (mg/mL)	Regressionequation	R^2	LOD (mg/mL)	LOQ (mg/mL)	Precision RSD (%)	Repeatability RSD (%)	Stability RSD(%)	Recovery (%)	Recovery RSD (%)
EGC	6.784 ± 0.038	0.010 – 1.000	y = 5393.1531x + 63.0632	0.9994	0.0028	0.00848	3.148	2.694	2.161	102.10 ± 2.12	2.076
Caffeine	8.913 ± 0.073	0.010 – 1.000	y = 68869.8404x + 986.3087	0.9964	0.0006	0.00182	2.448	2.161	1.945	103.15 ± 2.11	2.046
EC	11.527 ± 0.051	0.010 – 1.000	y = 18291.5295x − 14.6890	1.0000	0.0015	0.00455	4.414	1.798	2.068	103.37 ± 1.79	1.732
EGCG	14.806 ± 0.103	0.010 – 1.000	y = 41138.2881x − 450.3200	0.9998	0.0017	0.00515	3.784	3.735	1.800	100.05 ± 2.01	2.009
ECG	21.238 ± 0.112	0.010 – 1.000	y = 45951.4636x − 505.6144	0.9997	0.0019	0.00576	4.143	1.772	1.798	102.93 ± 2.07	2.011
L-theanine	4.695 ± 0.036	0.050 – 1.000	y = 9466.6180x + 99.1234	0.9994	0.0027	0.00818	4.650	2.191	1.982	97.49 ± 1.17	1.200

Table S9. Standardized values of seven chemical indicators.

Sample Code	Group	Z (EGC)	Z (Caffeine)	Z (EC)	Z (EGCG)	Z (ECG)	Z (L-Theanine)	Z (WEC)
A-F-S-L-1	AH-HWE	-0.0390	0.1712	-0.5124	0.2731	-0.4285	0.2766	-0.3668
A-F-S-L-2	AH-HWE	0.0943	-0.1807	-0.4881	0.7522	0.0319	0.5588	-0.1073
A-F-S-L-3	AH-HWE	0.7572	-1.0888	0.1452	2.0046	0.7491	0.8998	1.0085
A-F-S-L-4	AH-HWE	0.9792	-1.2305	0.3461	2.1562	0.9987	1.8745	2.0650
A-F-S-L-5	AH-HWE	0.2433	-0.9448	0.0721	1.4650	0.6304	0.6889	-0.0073
A-F-S-T-1	AH-HWE	-0.4672	-0.3084	-0.0984	0.2982	-0.1399	-0.3782	0.3803
A-F-S-T-2	AH-HWE	-0.1468	-0.3351	-0.0862	0.7585	0.2515	0.1716	0.9054
A-F-S-T-4	AH-HWE	0.9824	-1.2386	0.3705	2.1771	1.0111	1.8829	2.1025
A-F-S-T-5	AH-HWE	1.0236	-1.2642	0.4253	2.2000	1.0288	1.9039	2.1838
A-F-S-M-1	AH-HWE	-0.3276	0.4534	-0.5124	0.1500	-0.3117	-0.5083	1.6962
A-F-S-M-2	AH-HWE	0.4400	-0.9657	2.4711	1.0985	0.4976	0.9071	1.7993
A-F-S-M-4	AH-HWE	0.0847	-0.5848	1.1559	0.8510	0.3276	1.3153	1.6837
A-F-S-M-5	AH-HWE	-0.5369	-0.3514	-0.4820	0.6730	0.2585	-0.3824	1.4024
A-F-R	AH-HWE	0.9475	2.9038	0.2000	2.0136	0.8217	1.6238	2.0087
A-L-S-L-1	AH-CB	-0.3276	1.2222	-0.8717	-1.2205	-1.6911	-0.8356	-1.0044
A-L-S-L-2	AH-CB	0.1545	1.0561	-0.6829	-1.0509	-1.3635	-0.8031	-0.6637
A-L-S-L-3	AH-CB	0.4495	0.8506	-0.4942	-0.9403	-1.2024	-0.6268	-0.3668
A-L-S-L-4	AH-CB	1.2171	0.1840	2.5259	0.1118	-0.7986	1.2502	0.3928
A-L-S-L-5	AH-CB	0.0023	1.0654	-0.7864	-1.1148	-1.5371	-0.8052	-0.8888
A-L-S-T-1	AH-CB	0.7032	0.8738	-0.5003	-0.5933	-1.3051	-0.5251	-0.3824
A-L-S-T-2	AH-CB	0.7254	0.6241	-0.4942	-0.3465	-1.2201	-0.4621	-0.2199
A-L-S-T-4	AH-CB	1.2171	0.1724	2.5259	0.1146	-0.7986	1.2408	0.4146

A-L-S-T-5	AH-CB	1.2234	0.1584	1.9170	0.1160	-0.7969	1.2618	0.5115
A-L-S-M-1	AH-CB	0.5129	0.6241	-0.4150	-0.3465	-1.0023	-0.6268	-0.1417
A-L-S-M-2	AH-CB	0.9253	0.3698	0.9976	-0.1907	-0.8854	0.3542	0.0333
A-L-S-M-4	AH-CB	0.9126	0.6206	-0.3541	-0.3451	-0.8925	-0.5765	-0.0011
A-L-S-M-5	AH-CB	0.3512	1.1270	-0.7012	-0.7832	-1.3777	-0.7685	-0.1949
A-L-R	AH-CB	1.1853	1.4649	2.3920	0.0638	-0.8606	1.1254	0.4365
A-M-S-L-1	AH-EAE	0.6652	0.3512	-0.2750	-0.2262	-0.9899	-0.5198	-0.6668
A-M-S-L-2	AH-EAE	0.9633	0.2978	-0.1288	-0.0092	-0.9740	-0.2722	-0.4387
A-M-S-L-3	AH-EAE	1.1346	-0.1098	0.0417	0.1820	-0.3276	-0.2114	0.1552
A-M-S-L-4	AH-EAE	1.4042	-0.2109	1.1864	0.6278	-0.2125	2.3257	0.5209
A-M-S-L-5	AH-EAE	1.0807	0.0980	-0.0071	0.0840	-0.4551	-0.2271	0.0771
A-M-S-T-1	AH-EAE	0.2243	0.9307	-0.2080	-0.7185	-1.3883	0.7592	-1.4545
A-M-S-T-2	AH-EAE	1.1346	0.2432	1.3995	0.2717	-0.6676	1.8011	-0.0480
A-M-S-T-4	AH-EAE	1.4232	-0.2155	1.2107	0.6347	-0.2036	2.3352	0.5491
A-M-S-T-5	AH-EAE	1.4740	-0.2422	1.2473	0.6458	-0.1806	2.3509	0.5897
A-M-S-M-1	AH-EAE	0.2021	0.4069	-0.2932	-0.0092	-0.9899	-0.6709	-1.6045
A-M-S-M-2	AH-EAE	0.9221	0.3512	-0.2750	0.2634	-0.9244	-0.4369	-0.3261
A-M-S-M-4	AH-EAE	1.1346	-0.1435	0.0417	0.6278	-0.4462	2.3226	0.2771
A-M-S-M-5	AH-EAE	1.0807	-0.1098	-0.1288	0.5888	-0.4551	2.3068	0.2521
A-M-R	AH-EAE	1.3344	1.8551	0.8880	0.6000	-0.2497	2.3152	0.3896
S-F-S-L-1	SX-HWE	0.6144	-0.3793	0.9793	0.8037	0.7739	0.2524	-0.9544
S-F-S-L-2	SX-HWE	0.6430	-0.4699	1.0402	0.9720	1.0908	0.5000	-0.7731
S-F-S-L-3	SX-HWE	0.7540	-0.9100	1.2716	1.7424	1.6964	0.5703	-0.3011
S-F-S-L-4	SX-HWE	1.2202	-1.2537	1.7283	1.9824	2.0453	2.3268	1.8431
S-F-S-L-5	SX-HWE	1.0648	-1.2340	1.3325	1.7911	1.8948	0.9869	0.2240

S-F-S-T-1	SX-HWE	0.3924	-0.5825	0.7601	0.7703	1.2697	0.4476	0.5334
S-F-S-T-2	SX-HWE	0.6874	-0.9007	1.0889	1.3190	1.3016	0.8935	0.7553
S-F-S-T-4	SX-HWE	1.2234	-1.2688	1.7344	1.9914	2.0471	2.3394	1.8837
S-F-S-T-5	SX-HWE	1.2393	-1.3037	1.7831	2.0088	2.0949	2.3992	1.9712
S-F-S-M-1	SX-HWE	0.1894	-0.3224	0.9489	0.8232	0.9456	-0.1043	-0.1542
S-F-S-M-2	SX-HWE	0.5605	-0.6185	1.3325	1.0464	1.1546	0.1832	0.4772
S-F-S-M-4	SX-HWE	0.5986	-1.0168	1.3934	1.2800	1.5619	1.9438	0.5897
S-F-S-M-5	SX-HWE	0.2338	-0.5697	0.9489	0.9108	1.1369	-0.0645	0.4053
S-F-R	SX-HWE	1.2837	2.9955	1.8074	2.0797	2.2224	2.3373	1.9368
S-L-S-L-1	SX-CB	-0.7177	1.4591	-0.8351	-1.6169	-1.5017	-0.8892	-1.2763
S-L-S-L-2	SX-CB	-0.2420	1.2303	-0.2567	-1.3965	-1.2803	-0.5167	-1.0044
S-L-S-L-3	SX-CB	-0.0168	0.8599	-0.2384	-1.2108	-1.2679	-0.4915	-0.8481
S-L-S-L-4	SX-CB	0.3321	-0.2086	-0.0801	-1.0008	-0.3205	-0.4779	-0.1073
S-L-S-L-5	SX-CB	-0.4481	1.3313	-0.2567	-1.4055	-1.3688	-0.5670	-1.2357
S-L-S-T-1	SX-CB	-0.1785	1.1502	-0.6342	-1.2692	-1.5017	-0.9038	-1.1232
S-L-S-T-2	SX-CB	-0.0897	1.1177	-0.3602	-1.1795	-1.3972	-0.5712	-1.0325
S-L-S-T-4	SX-CB	0.3353	-0.2179	-0.0679	-0.9966	-0.3205	-0.4779	-0.0823
S-L-S-T-5	SX-CB	0.3417	-0.2260	-0.0497	-0.9939	-0.3099	-0.4737	-0.0042
S-L-S-M-1	SX-CB	-0.4006	1.2268	-0.5977	-1.6169	-1.5017	-0.7769	-0.7293
S-L-S-M-2	SX-CB	-0.0263	0.4627	-0.3724	-1.1573	-0.7473	-0.7139	-0.5731
S-L-S-M-4	SX-CB	0.0181	0.3802	-0.2993	-1.1294	-0.6446	-0.6489	-0.4512
S-L-S-M-5	SX-CB	-0.0358	0.5463	-0.4150	-1.1886	-0.7792	-0.6909	-0.6325
S-L-R	SX-CB	0.2021	1.8354	-0.1715	-1.0224	-0.3542	-0.5041	-0.2105
S-M-S-L-1	SX-EAE	0.6652	0.3617	0.6871	-0.4188	-0.8341	-1.1043	-1.3513
S-M-S-L-2	SX-EAE	0.7001	0.2618	0.6932	-0.2373	-0.6995	-0.7800	-1.1169

S-M-S-L-3	SX-EAE	1.0046	0.2176	0.7358	0.0930	-0.2178	-0.6111	-0.3074
S-M-S-L-4	SX-EAE	1.5691	-0.3979	1.4238	0.7286	0.3241	0.2042	0.4053
S-M-S-L-5	SX-EAE	1.3154	-0.2190	1.2838	0.4637	0.1629	-0.2764	-0.1667
S-M-S-T-1	SX-EAE	0.8333	0.1515	0.6810	0.1674	-0.0832	-0.2764	-0.3980
S-M-S-T-2	SX-EAE	1.0268	-0.0123	0.9915	0.2641	-0.0248	0.1433	-0.0792
S-M-S-T-4	SX-EAE	1.5850	-0.4153	1.4360	0.7328	0.3382	0.2178	0.4397
S-M-S-T-5	SX-EAE	1.5977	-0.4362	1.4543	0.7425	0.3630	0.2304	0.4803
S-M-S-M-1	SX-EAE	0.2243	0.3617	-0.1167	-0.4188	-0.8341	-1.1043	-1.8014
S-M-S-M-2	SX-EAE	0.6652	0.2618	0.6871	-0.2373	-0.6995	-0.7800	-1.1982
S-M-S-M-4	SX-EAE	1.0046	-0.3874	0.7358	0.4470	0.0337	-0.1201	0.2677
S-M-S-M-5	SX-EAE	0.7001	0.2176	0.6932	0.0930	-0.2178	-0.2764	0.2209
S-M-R	SX-EAE	1.4740	2.0363	1.3629	0.5200	0.2355	0.0510	0.2552
Y-F-S-L-1	YN-HWE	-1.2791	-0.0634	-0.8960	-0.5022	0.1913	-0.8262	-1.0638
Y-F-S-L-2	YN-HWE	-1.2506	-0.3119	-0.7986	-0.2519	0.5277	-0.7255	-0.6575
Y-F-S-L-3	YN-HWE	-1.1872	-1.0842	-0.5855	0.2870	1.3086	-0.7150	-0.4480
Y-F-S-L-4	YN-HWE	-1.0317	-1.7078	-0.4089	0.6890	1.8363	-0.5009	1.2805
Y-F-S-L-5	YN-HWE	-1.0666	-1.4895	-0.5307	0.6632	1.7956	-0.6950	0.7960
Y-F-S-T-1	YN-HWE	-1.2062	-0.9983	-0.6099	0.3823	1.3936	-1.0507	0.6553
Y-F-S-T-2	YN-HWE	-1.2030	-1.2410	-0.5429	0.5589	1.6380	-0.7255	0.8929
Y-F-S-T-4	YN-HWE	-1.0222	-1.7299	-0.3967	0.7001	1.8417	-0.4946	1.3367
Y-F-S-T-5	YN-HWE	-1.0127	-1.7485	-0.3846	0.7182	1.8788	-0.4758	1.4211
Y-F-S-M-1	YN-HWE	-1.2220	-1.1864	-1.0178	0.2523	1.4839	-0.7255	0.0364
Y-F-S-M-2	YN-HWE	-1.0666	-1.2712	-0.5855	0.2710	1.5566	-0.5324	0.2771
Y-F-S-M-4	YN-HWE	-1.5234	-1.1353	-1.1579	0.2335	1.1386	-0.7622	-0.2261
Y-F-S-M-5	YN-HWE	-1.6185	-0.9588	-1.2918	0.2314	1.0306	-0.7832	-0.3355

Y-F-R	YN-HWE	-0.9810	3.3985	-0.3176	0.7425	1.9320	-0.5009	1.3336
Y-L-S-L-1	YN-CB	-1.5139	0.3245	-1.3040	-1.4737	-0.8783	-0.9626	-0.9575
Y-L-S-L-2	YN-CB	-1.4028	0.2548	-1.2675	-1.4152	-0.8323	-0.9353	-0.5793
Y-L-S-L-3	YN-CB	-1.2411	0.1666	-1.1700	-1.3450	-0.7473	-0.9185	-0.3261
Y-L-S-L-4	YN-CB	-1.1491	-0.2887	-1.0422	-1.1350	-0.3365	-0.7926	0.5022
Y-L-S-L-5	YN-CB	-1.5234	0.6195	-1.4440	-1.6079	-1.1687	-1.1011	-1.1794
Y-L-S-T-1	YN-CB	-1.5931	0.0225	-1.2187	-1.3798	-0.6906	-0.9290	-0.4949
Y-L-S-T-2	YN-CB	-1.5551	-0.0541	-1.1579	-1.3269	-0.4746	-0.8724	-0.3136
Y-L-S-T-4	YN-CB	-1.1491	-0.2991	-1.0361	-1.1336	-0.3223	-0.7884	0.5209
Y-L-S-T-5	YN-CB	-1.1459	-0.3131	-1.0239	-1.1211	-0.2975	-0.7737	0.5897
Y-L-S-M-1	YN-CB	-1.4980	0.2142	-1.3588	-1.3193	-0.5224	-0.6730	-0.2574
Y-L-S-M-2	YN-CB	-1.3997	-0.1470	-1.1457	-1.2532	-0.4268	-0.8377	-0.0292
Y-L-S-M-4	YN-CB	-1.4409	-0.0913	-1.1700	-1.2762	-0.4285	-0.8545	-0.0605
Y-L-S-M-5	YN-CB	-1.5519	0.4766	-1.4379	-1.3262	-0.5189	-0.8850	-0.2636
Y-L-R	YN-CB	-1.1618	1.9805	-1.0543	-1.1767	-0.3630	-0.8052	0.3334
Y-M-S-L-1	YN-EAE	-1.3457	0.2060	-0.9691	-1.1635	-0.8040	-0.4044	-1.1794
Y-M-S-L-2	YN-EAE	-1.2823	-0.1760	-0.9143	-0.8687	-0.5100	-0.3310	-1.0732
Y-M-S-L-3	YN-EAE	-1.0571	-0.8368	-0.8717	-0.4202	0.0638	-0.2743	-0.5949
Y-M-S-L-4	YN-EAE	-0.8954	-1.1515	-0.7134	-0.0182	0.5631	0.0782	0.3459
Y-M-S-L-5	YN-EAE	-0.9207	-0.8798	-0.7377	-0.1566	0.3843	-0.0529	-0.0761
Y-M-S-T-1	YN-EAE	-1.3267	-0.8531	-0.9569	-0.5878	-0.0514	-0.8304	-0.7669
Y-M-S-T-2	YN-EAE	-1.2823	-0.9123	-0.8534	-0.3228	0.2550	-0.3310	-0.4293
Y-M-S-T-4	YN-EAE	-0.8858	-1.1817	-0.6829	-0.0134	0.5826	0.0845	0.3646
Y-M-S-T-5	YN-EAE	-0.8763	-1.2247	-0.6220	-0.0043	0.6198	0.0992	0.4709
Y-M-S-M-1	YN-EAE	-1.4187	0.4209	-1.5171	-1.1322	-0.8075	-1.0350	-4.4020

Y-M-S-M-2	YN-EAE	-1.3806	0.0086	-1.3527	-0.7679	-0.2692	-0.7800	-3.1705
Y-M-S-M-4	YN-EAE	-0.9651	-0.8914	-0.7377	-0.1643	0.3489	-0.2260	-0.0730
Y-M-S-M-5	YN-EAE	-1.0635	-0.7288	-0.9569	-0.2143	0.3117	-0.4233	-0.1730
Y-M-R	YN-EAE	-0.9112	2.7969	-0.7864	-0.0363	0.4498	0.0279	0.2459

Note: Values are Z-score standardized values. In the extraction-process code, F, M, and L represent hot-water extraction (HWE), enzyme-assisted extraction (EAE), and cold brewing (CB), respectively. In the experiment-type code, S and R represent single-factor experiments and optimized response surface conditions, respectively. For single-factor experiments, L, T, and M represent solid-to-liquid ratio, extraction time, and particle size, respectively; numbers 1–5 indicate the first to fifth levels of each single factor.

Table S10. Major Component Scores and Comprehensive Chemical Scores for All Samples.

**Sample code**	**F1**	**F2**	**F**	**Rank**	**Score zone**
S-F-R	2.1282	1.2685	1.8652	1	High-score zone
S-F-S-T-5	2.4917	-0.5101	1.5732	2	High-score zone
S-F-S-T-4	2.4322	-0.4911	1.5377	3	High-score zone
S-F-S-L-4	2.4156	-0.4822	1.5289	4	High-score zone
A-F-R	1.3353	1.1043	1.2646	5	High-score zone
A-M-S-T-5	1.3065	1.0704	1.2343	6	High-score zone
A-M-R	0.9426	1.8600	1.2233	7	High-score zone
A-M-S-T-4	1.2691	1.0653	1.2068	8	High-score zone
A-L-R	0.7767	2.1322	1.1915	9	High-score zone

A-M-S-L-4	1.2493	1.0599	1.1914	10	High-score zone
A-L-S-L-4	0.9697	1.6349	1.1733	11	High-score zone
A-L-S-T-4	0.9737	1.6254	1.1731	12	High-score zone
A-F-S-T-5	1.9579	-0.6461	1.1611	13	High-score zone
A-F-S-T-4	1.9073	-0.6487	1.1252	14	High-score zone
A-F-S-L-4	1.8843	-0.6422	1.1112	15	High-score zone
A-F-S-M-2	1.5989	-0.0026	1.1088	16	High-score zone
A-L-S-T-5	0.8728	1.4526	1.0502	17	High-score zone
S-F-S-M-4	1.6241	-0.3463	1.0211	18	High-score zone
A-M-S-T-2	0.8092	1.4100	0.9930	19	High-score zone
S-F-S-L-5	1.6120	-0.5335	0.9555	20	High-score zone
S-M-R	0.5884	1.6286	0.9067	21	High-score zone
S-M-S-T-5	1.0179	0.5830	0.8848	22	High-score zone
S-M-S-T-4	0.9923	0.5962	0.8711	23	High-score zone
S-M-S-L-4	0.9720	0.6027	0.8590	24	High-score zone
A-M-S-M-4	0.8652	0.8063	0.8471	25	High-score zone
S-F-S-T-2	1.3299	-0.4213	0.7940	26	High-score zone
A-M-S-M-5	0.7973	0.7631	0.7868	27	High-score zone
A-F-S-M-4	1.2022	-0.1606	0.7852	28	High-score zone
S-F-S-L-3	1.2716	-0.4432	0.7469	29	High-score zone
A-F-S-L-3	1.2837	-0.6085	0.7047	30	High-score zone
S-F-S-M-2	1.0282	-0.2716	0.6304	31	High-score zone
S-M-S-L-5	0.5686	0.6375	0.5897	32	High-score zone
S-M-S-T-2	0.4661	0.6739	0.5297	33	High-score zone
S-F-S-L-2	0.7596	-0.0025	0.5264	34	High-score zone

A-L-S-M-2	0.2218	1.1824	0.5157	35	High-score zone
S-F-S-T-1	0.8975	-0.4583	0.4826	36	High-score zone
S-M-S-M-4	0.5086	0.3146	0.4493	37	High-score zone
S-F-S-L-1	0.5461	0.1344	0.4201	38	High-score zone
S-F-S-M-5	0.7804	-0.4861	0.3929	39	High-score zone
A-F-S-L-5	0.7635	-0.5632	0.3575	40	High-score zone
S-F-S-M-1	0.5748	-0.2467	0.3235	41	High-score zone
S-M-S-M-5	0.2162	0.5519	0.3189	42	High-score zone
S-M-S-T-1	0.1617	0.6005	0.2960	43	Medium-score zone
S-M-S-L-3	0.0996	0.7076	0.2857	44	Medium-score zone
A-M-S-L-3	0.1898	0.4676	0.2748	45	Medium-score zone
A-M-S-L-5	0.0857	0.5877	0.2393	46	Medium-score zone
A-F-S-T-2	0.4341	-0.4903	0.1512	47	Medium-score zone
A-M-S-L-2	-0.2050	0.8647	0.1223	48	Medium-score zone
A-F-S-L-2	0.2220	-0.1544	0.1068	49	Medium-score zone
A-M-S-M-2	-0.1867	0.7486	0.0995	50	Medium-score zone
Y-F-R	0.1219	-0.0980	0.0546	51	Medium-score zone
S-M-S-L-2	-0.3303	0.8968	0.0452	52	Medium-score zone
S-M-S-M-2	-0.3539	0.8927	0.0276	53	Medium-score zone
A-L-S-M-4	-0.3388	0.8191	0.0155	54	Medium-score zone
A-M-S-T-1	-0.6298	1.3346	-0.0287	55	Medium-score zone
A-F-S-L-1	-0.1504	0.1510	-0.0582	56	Medium-score zone
A-L-S-T-2	-0.4774	0.8817	-0.0616	57	Medium-score zone
S-M-S-L-1	-0.5330	0.9775	-0.0708	58	Medium-score zone
A-M-S-L-1	-0.4490	0.7540	-0.0809	59	Medium-score zone

A-F-S-M-1	0.0003	-0.2750	-0.0840	60	Medium-score zone
A-F-S-M-5	0.2406	-0.8975	-0.1076	61	Medium-score zone
A-L-S-T-1	-0.6270	1.0460	-0.1150	62	Medium-score zone
A-L-S-M-1	-0.4810	0.7009	-0.1193	63	Medium-score zone
S-L-R	-0.6340	0.9984	-0.1345	64	Medium-score zone
A-F-S-T-1	-0.0341	-0.4230	-0.1531	65	Medium-score zone
S-L-S-T-5	-0.3318	0.1717	-0.1777	66	Medium-score zone
Y-M-R	-0.4579	0.4186	-0.1897	67	Medium-score zone
S-L-S-T-4	-0.3565	0.1824	-0.1916	68	Medium-score zone
S-L-S-L-4	-0.3664	0.1857	-0.1975	69	Medium-score zone
A-L-S-L-3	-0.7543	0.9149	-0.2435	70	Medium-score zone
A-M-S-M-1	-0.7065	0.6765	-0.2833	71	Medium-score zone
A-L-S-M-5	-0.8299	0.9461	-0.2864	72	Medium-score zone
Y-F-S-T-5	0.5835	-2.2858	-0.2945	73	Medium-score zone
Y-F-S-T-4	0.5457	-2.2602	-0.3129	74	Medium-score zone
Y-F-S-L-4	0.5232	-2.2478	-0.3247	75	Medium-score zone
S-L-S-L-3	-0.9268	0.9381	-0.3562	76	Medium-score zone
Y-M-S-T-5	0.0509	-1.3104	-0.3657	77	Medium-score zone
S-L-S-M-4	-0.7162	0.4113	-0.3712	78	Medium-score zone
Y-M-S-T-4	-0.0007	-1.2843	-0.3935	79	Medium-score zone
S-M-S-M-1	-0.8690	0.6565	-0.4022	80	Medium-score zone
Y-M-S-L-4	-0.0212	-1.2737	-0.4045	81	Medium-score zone
S-L-S-T-2	-1.0583	1.0464	-0.4143	82	Medium-score zone
S-L-S-M-2	-0.8104	0.4587	-0.4220	83	Medium-score zone
A-L-S-L-2	-1.0180	0.9233	-0.4239	84	Medium-score zone

S-L-S-M-5	-0.8486	0.5058	-0.4342	85	Low-score zone
Y-F-S-L-5	0.3187	-2.1445	-0.4350	86	Low-score zone
S-L-S-L-2	-1.0885	1.0411	-0.4369	87	Low-score zone
Y-F-S-T-2	0.2288	-2.0445	-0.4668	88	Low-score zone
Y-M-S-L-5	-0.2338	-1.0549	-0.4851	89	Low-score zone
Y-F-S-M-2	0.0823	-1.8429	-0.5068	90	Low-score zone
S-L-S-L-5	-1.2090	1.0615	-0.5142	91	Low-score zone
A-L-S-L-5	-1.1586	0.9430	-0.5155	92	Low-score zone
Y-M-S-M-4	-0.2863	-1.0887	-0.5318	93	Low-score zone
Y-F-S-T-1	-0.0132	-1.8653	-0.5799	94	Low-score zone
S-L-S-T-1	-1.2662	0.9664	-0.5830	95	Low-score zone
S-L-S-M-1	-1.2830	0.9180	-0.6095	96	Low-score zone
Y-F-S-L-3	-0.1803	-1.6454	-0.6286	97	Low-score zone
A-L-S-L-1	-1.3315	0.9370	-0.6373	98	Low-score zone
Y-M-S-M-5	-0.4468	-1.1118	-0.6503	99	Low-score zone
Y-M-S-L-3	-0.5625	-0.9389	-0.6777	100	Low-score zone
Y-L-R	-1.0704	0.1909	-0.6844	101	Low-score zone
Y-F-S-M-1	-0.1515	-1.9463	-0.7007	102	Low-score zone
Y-M-S-T-2	-0.5137	-1.1681	-0.7139	103	Low-score zone
Y-F-S-L-2	-0.6194	-1.0241	-0.7432	104	Low-score zone
Y-L-S-T-5	-0.7672	-0.8239	-0.7845	105	Low-score zone
Y-L-S-T-4	-0.7959	-0.8044	-0.7985	106	Low-score zone
S-L-S-L-1	-1.5437	0.8881	-0.7996	107	Low-score zone
Y-L-S-L-4	-0.8056	-0.7941	-0.8021	108	Low-score zone
Y-M-S-L-2	-0.9894	-0.4412	-0.8217	109	Low-score zone

Y-F-S-M-4	-0.3640	-1.9163	-0.8390	110	Low-score zone
Y-F-S-L-1	-0.8908	-0.7624	-0.8515	111	Low-score zone
Y-M-S-L-1	-1.2080	-0.1753	-0.8920	112	Low-score zone
Y-F-S-M-5	-0.4710	-1.8609	-0.8963	113	Low-score zone
Y-M-S-T-1	-0.8445	-1.0715	-0.9140	114	Low-score zone
Y-L-S-M-2	-1.0448	-0.7477	-0.9539	115	Low-score zone
Y-L-S-L-3	-1.2063	-0.3992	-0.9593	116	Low-score zone
Y-L-S-M-4	-1.0782	-0.7421	-0.9753	117	Low-score zone
Y-L-S-M-1	-1.1820	-0.5915	-1.0013	118	Low-score zone
Y-L-S-T-2	-1.1739	-0.7136	-1.0330	119	Low-score zone
Y-L-S-L-2	-1.3495	-0.3797	-1.0527	120	Low-score zone
Y-L-S-M-5	-1.2814	-0.5532	-1.0586	121	Low-score zone
Y-L-S-T-1	-1.2997	-0.6082	-1.0881	122	Low-score zone
Y-L-S-L-1	-1.4867	-0.3323	-1.1334	123	Low-score zone
Y-M-S-M-2	-1.5656	-0.3930	-1.2068	124	Low-score zone
Y-L-S-L-5	-1.7032	-0.1142	-1.2170	125	Low-score zone
Y-M-S-M-1	-2.1293	0.0957	-1.4484	126	Low-score zone

Note: In the extraction-process code, F, M, and L represent hot-water extraction, enzyme-assisted extraction, and cold brewing, respectively. In the experiment-type code, S and R represent single-factor experiments and optimized response surface conditions, respectively. For single-factor experiments, L, T, and M represent solid-to-liquid ratio, extraction time, and particle size, respectively; numbers 1–5 indicate the first to fifth levels of each single factor.

Table S11. Liquid phase gradient setting parameters.

Time (min)	Flow (mL/min)	B (%)
0.0-5.0	0.40	5-65
5.0-6.0	0.40	65-99
6.0-7.5	0.40	99
7.5-7.6	0.40	99-5
7.6-10.0	0.40	5

Table S12. Mass spectrum parameters.

Parameter	Positive ion mode	Negative ion mode
Acquisition time (min)	10	10
IonSpray voltage (V)	5000	-4000
Source temperature (°C)	550	550
Ion Source Gas 1 (psi)	50	50
Ion Source Gas 2 (psi)	60	60
Curtain Gas (psi)	35	35
Declustering potential (V)	80	-80
MS1 collision energy (V)	10	-10
MS2 collision energy (V)	30	-30
Collision energy spread (V)	15	15
MS1 TOF mass range (Da)	50-1250	50-1250
MS2 TOF mass range (Da)	25-1250	25-1250

Table S13. Differential metabolites and identification results.

Scan patterns	All	MS/MS-supported identification	KEGG	HMDB
pos	6505	1685	709	1024
neg	9392	2464	988	1581
